# Effect of photobiomodulation combined with physical therapy on functional performance in children with myelomeningocele: A protocol randomized clinical blind study

**DOI:** 10.1371/journal.pone.0253963

**Published:** 2021-10-06

**Authors:** Tamiris Silva, Juliana Roque de Queiroz, Karina Helga Leal Turcio, Daysi da Cruz Tobelem, Tamires Ribeiro Araújo, Kevelin Siqueira Lira Coutinho, Maria Cristina Chavantes, Anna Carolina Ratto Tempestini Horliana, Alessandro Melo Deana, Daniela de Fátima Teixeira da Silva, Paula Midori Castelo, Kristianne Porta Santos Fernandes, Lara Jansiski Motta, Raquel Agnelli Mesquita-Ferrari, Sandra Kalil Bussadori

**Affiliations:** 1 Universidade Nove de Julho, UNINOVE, São Paulo, SP, Brazil; 2 Universidade Estadual de São Paulo (UNESP), Aracatuba, SP, Brazil; 3 Universidade Federal de São Paulo (UNIFESP), Diadema, SP, Brazil; Prince Sattam Bin Abdulaziz University, College of Applied Medical Sciences, SAUDI ARABIA

## Abstract

**Introduction:**

Myelomeningocele is a severe type of spina bifida, resulting from improper closure of the neural tube. This condition drastically affects the structures of the spinal cord resulting in deficiencies. The combination of these deficiencies results in an overall decrease in mobility and functional participation amongst this population. Physiotherapy plays an essential role in rehabilitating people with MMC. The current literature shows that resources such as photobiomodulation (PBM) may support the rehabilitation of neurological conditions. The aim of the proposed study is to evaluate the effects of photobiomodulation (PBM) combined with physical therapy on functional performance in children with low lumbosacral myelomeningocele.

**Materials and methods:**

This is a protocol randomized clinical blind study, that will include 30 individuals of both sexes, aged between 5 to 8 years, diagnosed with low and sacral lumbar myelomeningocele and capable of performing the sit-to-stand task. The participants will be randomly assigned into two treatment groups: PBM + physiotherapeutic exercises and sham PBM + physiotherapeutic exercises. Irradiation will be carried out with light emitting diode (LED) at a wavelength of 850 nm, energy of 25 J per point, 50 seconds per point and a power of 200 mW. The same device will be used in the placebo group but will not emit light. Muscle activity will be assessed using a portable electromyograph (BTS Engineering) and the sit-to-stand task will be performed as a measure of functioning. Electrodes will be positioned on the lateral gastrocnemius, tibialis anterior and rectus femoris muscles. The Pediatric Evaluation of Disability Inventory will be used to assess functional independence. Quality of life will be assessed using the Child Health Questionnaire—Parent Form 50. Changes in participation will be assessed using the Participation and Environment Measure for Children and Youth. The data will be analyzed with the aid of GraphPad PRISM.

**Discussion:**

The results of this study can contribute to a better understanding of the effectiveness of PBM on functioning and quality of life in children with myelomeningocele.

**Clinical trial registration:**

**ClinicalTrials.gov Identifier**: NCT04425330.

## Introduction

Myelomeningocele (MMC) is a severe type of spina bifida resulting from the improper closure of the neural tube [1]. The multifactor etiology involves environmental and maternal factors. This condition drastically affects the structures of the spinal cord, which can result in paraplegia, skeletal deformities, muscle weakness, the loss of sensation, impaired coordination, impaired balance, hydrocephalus, Arnold Chiari malformation, and defecatory, urinary and sexual dysfunctions, reducing the mobility and functionality of these individuals [2]. After an injury to the central nervous system (CNS), most axons do not recover due to regenerative failure, which usually results in permanent disability [3]. The aim of treatment strategies for this condition is to minimize the extent of the defect by reducing possible sequelae. Classic treatment for MMC consists of surgical closure of the MMC defect in the intrauterine phase or shortly following birth [3].

Physiotherapeutic interventions focus on the optimization of mobility and maximization of independence and participation, which can be facilitated by muscle strengthening, adaptive positioning and better postural control. However, evidence on the effectiveness of physiotherapeutic exercises in children with MMC is limited [4].

The current literature describes resources that use light as a therapeutic means to assist in the rehabilitation of patients with neurological conditions, such as stroke, neurodegenerative diseases and spinal cord injuries [5]. Photobiomodulation (PBM) is the application of a low intensity light (red and infrared light), such as low-level laser or a light emitting diode (LED), to biological tissues [6,7]. Both laser and LED have monochromatic radiation, although LED has a larger spectral width. Furthermore, Laser waves have organization favoring greater collimation, whereas LED waves are incoherent and the light is therefore not collimated, which enables treating larger areas. Despite some differences in the mode of functioning, both devices are efficient for PBM and LED has the advantage of being more affordable [8,9].

The therapeutic effect of phototherapy is achieved through the application of monochromatic or narrow-band light in tissues. PBM influences cell activity through the stimulation or inhibition of chemical and physiological functions [5]. The magnitude of the effect of PBM is influenced by the wavelength, energy density (fluence), power density, type of injury and absorption spectrum of the photoreceptor. Photons stimulate chemical changes in the interior of the cell, causing biological reactions, triggering neuroprotective responses, improving metabolism and blood flow as well as diminishing inflammatory processes and oxidative stress. As a result of these benefits, PBM has been widely used as an adjuvant in the treatment of numerous diseases [5,10,11].

Studies involving an experimental model of spinal cord injury have demonstrated that both the red and infrared wavelengths have the potential to be an effective, non-invasive means of therapy, promoting axonal sprouting, an increased concentration of glial cells and nerve connections as well as improvements in function and sensitivity [12,13]. In a clinical trial involving individuals with spinal cord injuries, PBM exerted positive effects on motor function, especially during isotonic contraction of the stimulated muscles, which were assessed using electromyography (EMG) [14]. Thus, the combination of physical therapy and PBM is promising treatment for individuals with MMC.

Therefore, the aim of the proposed study is to evaluate the effects of photobiomodulation combined with physical therapy on the functional performance of children with low lumbosacral myelomeningocele. We hypothesise that a photobiomodulation combined with physical therapy may enhance functional performance and health-related quality of life of children with MMC.

## Materials and methods

Invitations to participate in the study will be made after the determination of patients undergoing physical therapy or on the waiting list of the Integrated Health Clinic of Nove de Julho University. The study will obey the norms governing research involving human subjects (Resolutions 466/12 and 510/2016 of the Brazilian National Board of Health) following approval from the Human Research Ethics Committee of Nove de Julho University (certificate number: 4.308.134). The protocol will be in accordance with the SPIRIT statement. The participants or their guardians will sign a statement of informed consent agreeing to participate in the study. Clinical trial registry: Trials (ClinicalTrials.gov Identifier: NCT04425330).

### Participants

Eligibility for participation in the study will depend on the following criteria:

Inclusion criteria: Individuals of both sexes, aged between 5 to 8 years, diagnosed with low and sacral lumbar myelomeningocele and capable of performing the sit-to-stand task (transferring from a sitting position to a standing position).

Exclusion criteria: Cognitive impairment that compromises the ability to communicate and answer the questions that will be posed; neuromuscular scoliosis; subluxation or luxation of the hip or knee; other disease of the central nervous system.

### Randomization

Participants will be assigned to a treatment group through a web-based randomization service (randomization.com) by an independent assessor, who, apart from generating the randomization sequence, is neither involved in the research process nor with participants. Both participants and main researchers will be masked to the randomization sequence throughout the whole study period. Participants will be randomized and assigned in two groups: Group 1 will be submitted to physiotherapeutic exercises and active PBM (n = 15). Group 2 will be submitted to physiotherapeutic exercises and sham PBM (n = 15).

### Blinding

- The participants will be unaware of whether they are in the active or sham PBM group.
- The evaluator and researcher in charge of the exercises will be unaware of the allocation of the participants to the different groups.
- A therapist who will not participate in the evaluations or physical therapy sessions will administer the photobiomodulation.

### Interventions

#### Photobiomodulation protocol

**Fig 1:**
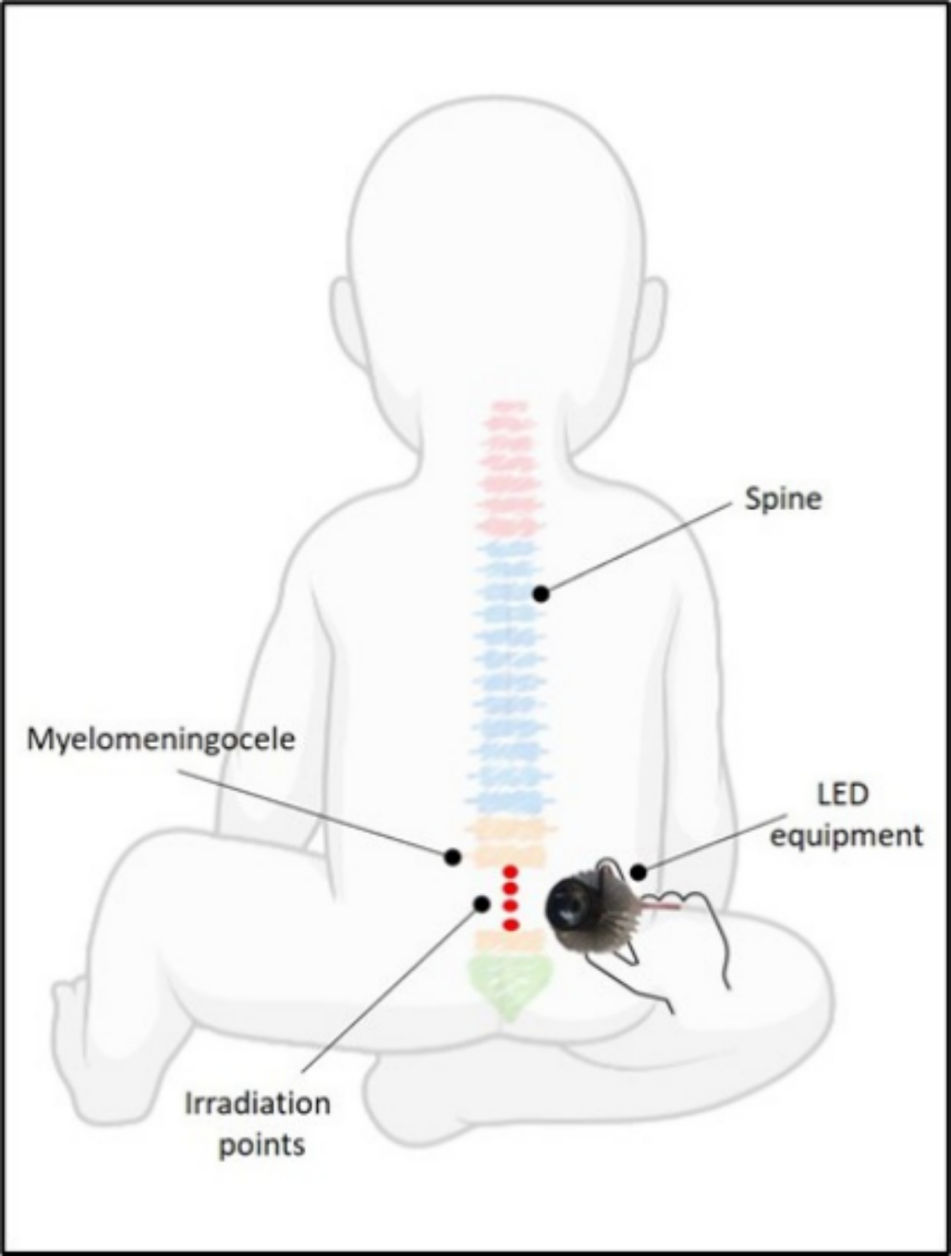
Participant positioning for irradiation.

For irradiation, the subjects will be positioned comfortably in the prone position on an examining table. To determine the irradiation site, palpation will be performed of the spinous processes for the localization of the defect. The location with the absence of a spinous process will be considered the defect site. The location with the absence of a spinous process will be considered the defect site, to which four points will be irradiated in sequence under the injury level (Fig 1).

The irradiation will be carried out twice a week for 3 minutes and 20 seconds in a total of 24 sessions with LED (Table 1).

**Table 1:** Photobiomodulation.

Parameter	
Center wavelength [nm]	850
Spectral bandwidth (FWHM) [nm]	20
Operating mode	Continuous wave
Average radiant power [mW]	500
Polarization	Random
Aperture diameter [cm]	1.9
Irradiance at aperture [mW/cm^2^]	176
Beam profile	Multimode
Beam spot size at target [cm^2^]	2.84
Irradiance at target [mW/cm^2^]	176
Exposure duration per site [s]	50
Radiant exposure [J/cm^2^]	9
Radiant energy per site [J]	25
Number of sites irradiated	4
Radiant energy per session [J]	100
Area irradiated per session [cm^2^]	11.34
Application technique	Contact
Number of treatment sessions	24
Frequency of treatment sessions	2x per week
Total radiant energy [J]	2400

nm = nanometer; J = joules; s = seconds; mW = miliwatts.

#### Physiotherapy

One of the characteristics of the physical therapy program is the practice of functional activities through repeated active, self-generated movements, which will be directly linked to the fulfillment of a goal defined by the child and/or family, such as walking without assistance during recess at school. Thus, the exercises will be individualized and personalized for each child [15].

Physiotherapy will be performed with the aim of minimizing disabilities and accelerating functional independence. According to Luft [16], it is necessary to establish the intensity, motivation and training elements in order for new motor representations and new sensory and motor connections to occur.

**Fig 2:**
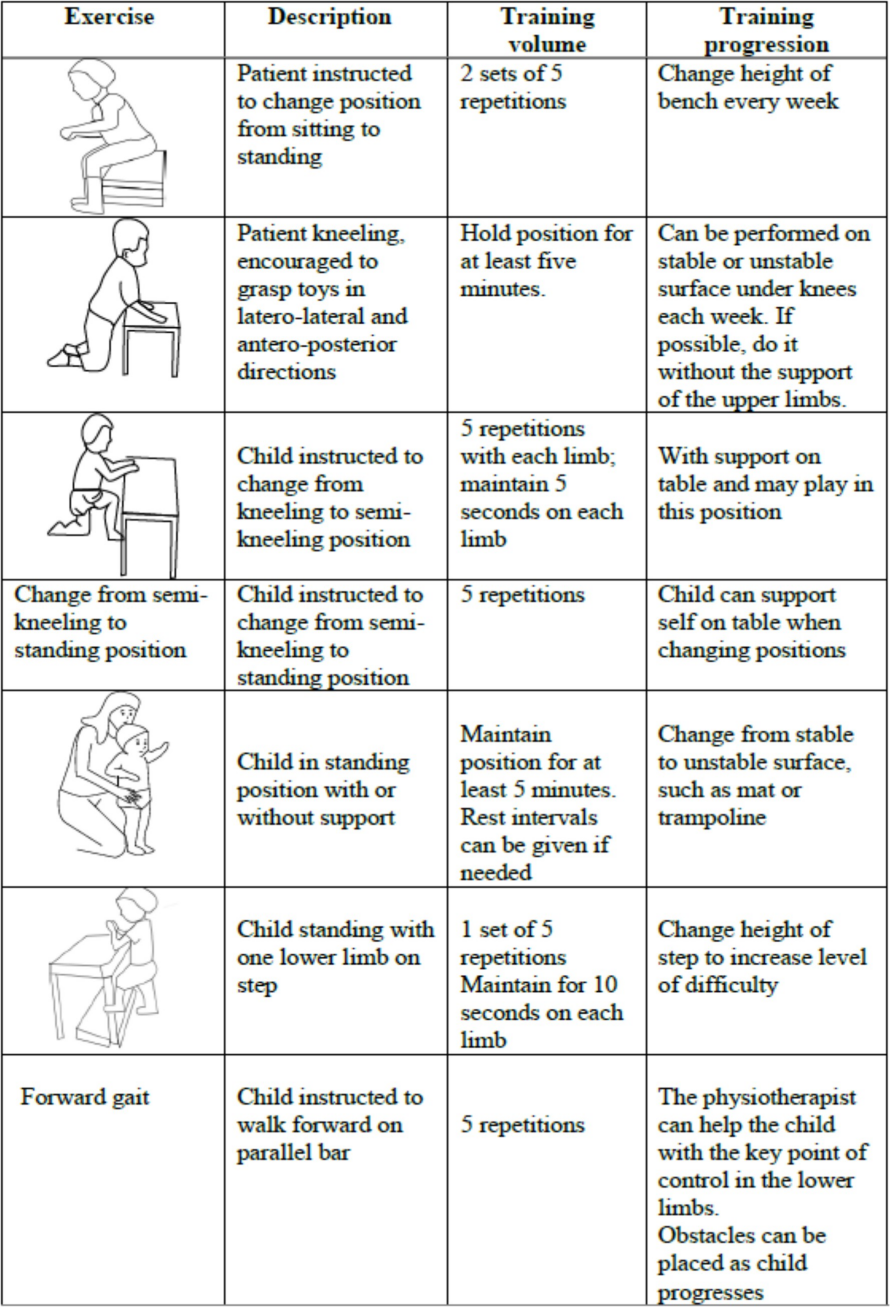
Functional training.

- Intensity: The participants in both groups will undergo the exercise program for 45 to 60 minutes in two sessions per week for 12 consecutive weeks.
- Motivation: All exercises will be associated with playful activities, such as ball playing, fishing, etc. The participants may also be take part in a story of princesses and superheroes that enable them to perform the tasks related to the goals [17].
- Training elements:

The exercises will be performed through the practice of functional training. (Fig 2) Five repetitions of each transition will be performed in each session. The participants will perform strengthening exercise of the lower limb muscles such as knee flexors and extensors and hip adductors and abductors (10 to 20 repetitions). Strengthening the trunk muscles will be performed through functional movements, such as transferring from the lying to sitting position, along with the aid of balls and rolls, including five repetitions in every session. The exercises will be performed with repetitions to promote motor learning as well as improve strength and endurance [4,18,19]. During each session, the participants will be given rest interval between sets.

#### Criteria for discontinuing intervention

Patients who fail to attend two consecutive sessions or three non-consecutive sessions will discontinue the intervention.

Materials used: Wedges, rolls, medicine balls, steps, balance bean, ankle weights (0.5–1.0 kg), cones, parallel bar, stools of different sizes, mats with different textures, trampoline, walkers.

#### Adherence

All interventions will be carried out individually. To ensure adherence to the predefined protocol, the physiotherapists will be instructed by the main investigator on how to perform the interventions and will receive exercise guidelines. The therapists will record details of the training in terms of the type and execution of exercises, level of intensity and repetitions. The physiotherapist in charge of the intervention at the participating physical therapy clinic will record symptoms of possible adverse events related to treatment. Any adverse events will be reported to the main investigator, who will then report to the regional ethics committee.

### Outcome measures

**Fig 3:**
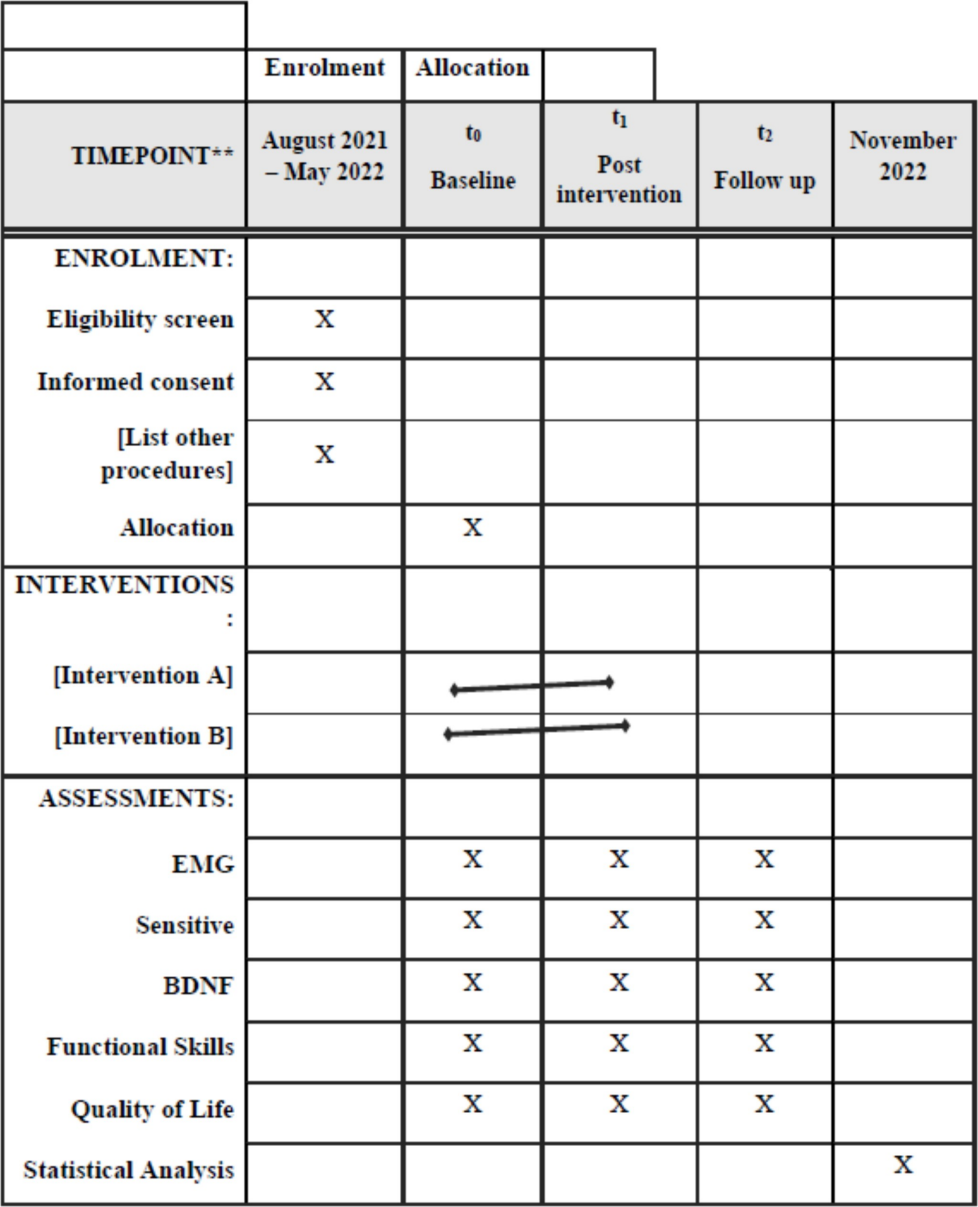
SPIRIT statement.

Evaluations will be performed prior to therapy (pre-intervention [T0]), after 24 sessions of PBM + physical therapy (post-intervention [T1]) and 30 days after the completion of the intervention (follow-up [T2]) (Fig 3).

#### Functional skills

The Pediatric Evaluation of Disability Inventory (PEDI) will be used for the assessment of functional independence. The PEDI version validated to Brazilian Portuguese will be used in this study [20]. PEDI is a questionnaire that can be administered in interview form to the parents or caregivers of the children and has three components: I–functional skills (197 items); II–caregiver assistance (20 items); and III–modifications of the environment (20 items). Part I addresses the child’s capacity to perform activities and tasks of daily living. Part II addresses the amount of assistance the caregiver must provide in order for the child to perform activities and tasks of daily living. Part III addresses the number of modifications of the environment used by the child. Each part is subdivided into three domains: self-care, mobility and social function [9]. In the proposed study, only Part I of the mobility domain will be used, which is composed of 65 items. Each item is attributed a score of either 0 (unable to perform) or 1 (able to perform). The total score is obtained by the sum of the items. For the caregiver assistance part, the items will be scored from 0 (completely dependent) to 5 (completely independent) points. The PEDI will be scored according to the instruction manual [21].

#### Quality of life

Quality of life will be assessed using the Child Health Questionnaire—Parent Form 50, which is a useful tool that enables the assessment of a child’s quality of life from the parents’ perspective. This questionnaire has been translated, cross-culturally adapted and validated for the Brazilian Portuguese language [22] and was designed for children five years of age or older, addressing their physical, emotional and social wellbeing from the perspective of parents or caregivers. The questionnaire is composed of 50 items distributed among 15 domains. The final score of each scale ranges from 0 to 100, with higher scores denoting better function or sensation (better quality of life) [23].

#### Participation and environment measure

Changes in participation will be assessed using the Participation and Environment Measure for Children and Youth (PEM-CY), which is a parental report instrument that addresses participation and environmental factors that affect the participation of children in three environments: home (13 items), school (16 items) and community (17 items). For each activity, the parent/caregiver will be asked to provide information on the frequency with which the child participates in one or more activities of this type. The response options are scored on an eight-point scale ranging from never to daily. The parents/caregivers will also be asked to rate the children’s involvement in each activity on a five-point scale from minimally involved to very involved and asked if they wished that their children’s participation changed in this type of activity. The maximum score is 122 points [24]. The PEM-CY version adapted to the Brazilian Portuguese will be used in this study [25].

#### Surface electromyography

Muscle activity will be evaluated using a portable electromyograph (BTS Engineering) synchronized to the BST EMG system. The sit-to-stand activity will be used as the functional measure [26,27]. Electrodes will be placed on the lateral gastrocnemius (LG), tibialis anterior (TA) and rectus femoris (RF) muscles. The preparation of the skin and placement of the electrode will follow the SENIAM recommendations for surface electromyography [28]. The electrodes will be placed with the child in prone position (LG) and supine position (TA and RF). We selected these superficial muscles for surface electromyography as they are deemed primary muscles of the gait cycle [29].

For the evaluation of the sit-to-stand test, the participant will be seated on a chair with hips, knees and ankles flexed at 90° and feet supported on the floor. The child will first be analyzed in the resting position and will then be instructed to stand up, remain in the standing position for 10 seconds and sit down again. The sit-to-stand test will be performed three times with a five-minute interval between tests. The children will perform the movement at the velocity with which they normally do so in their daily routine.

#### Calculation of sample size and statistical analysis

The sample size was calculated using the G * Power software (version 3.1.9.2) based on our previous pilot study (F test, repeated-measures ANOVA, intra-between interactions). Considering an effect size of 0.58 for the primary outcome (functional skills) and assuming a possible 10% dropout rate, a total of 30 patients (15 in each group) will be required to reach a 5% significance level with a 90% power. Descriptive statistics will be performed to determine the normality of the data and the mean change (standard deviation, 95% confidence interval) in each group will be calculated. GraphPad PRISM version 7.0 (GraphPad Software—San Diego, CA, USA) will be used for all analyses. A mixed factor ANOVA model will be employed to compare the effects of the interventions in each group in terms of functional variables (PEDI and EMG), quality of life questionnaire and social participation over time (pre-treatment, post-treatment and 30-day follow-up). A 95% confidence interval will be used to establish any differences and a p-value <0.05 will be indicative of a statistically significant difference between groups (two-tailed). If the supposition of normality is not met, pairwise comparisons between groups will be performed using a nonparametric test, as appropriate, for independent samples (Mann-Whitney U test) or dependent samples (Wilcoxon test). The alpha level will be adjusted to 0.0033 (Bonferroni correction) to avoid a type 1 error.

## Discussion

This randomized clinical study will expand our understanding of how physical therapy combined with photobiomodulation can affect functional performance, participation and quality of life in children with low lumbosacral myelomeningocele. Thus, the study will assist in optimizing future rehabilitation interventions for this population. Moreover, few studies have evaluated the effects of rehabilitation in children with myelomeningocele.

The proposed protocol is expected to improve functional performance, participation and quality of life in these children. In the rehabilitation process for children with MMC with the aim of achieving independence on activities of daily living, it is necessary for healthcare providers to have a holistic view of the child and his/her disability [20]. Individuals with MMC are known to have difficulties in functional participation, mainly concerning self-care, housing, and functional mobility, leading to diminished quality of life [30]. Physical therapists have used several outcome measures to evaluate gross motor function in children with MMC, such as the PEDI questionnaires, based on observing the children’s abilities [26]. The sit-to-stand task is an accessible tool applied to evaluate the functional strength of the lower limbs. The ability to rise from a seated position is one of the most fundamental movements among normal daily living activities, such as walking or going to the bathroom [31]. Among children with MMC, the sit-to-stand task is a reliable outcome measure, which has been examined regarding reliability and concurrent validity in children with MMC [32]. On the other hand, sit-to-stand activity is a complex activity that should be investigated in terms of its electromyographic features [33].

Animal studies have shown that the specific training of tasks and repetitive exercises are key to the promotion of synaptogenesis and have demonstrated that the acquisition and transference of skills to other activities are more effective when specific tasks are performed that are relevant to the context in comparison to passive modalities. Moreover, a study involving an experimental spinal cord injury model showed that exercise stimulated the structural and physiological plasticity of motor neurons below the level of the injury and increased post-exercise brain-derived neurotrophic factor levels, which was associated with motor recovery in animals [34,35]. However, physical training in humans may be limited, as restrictions regarding certain subsystems may impede the proper practice of tasks. The solution to these problems involves the strengthening of an appropriate training environment and the understanding of the interdependence between the body and nervous system. Moreover, the multiple factors that contribute to motor, sensory and cognitive functions are fundamental to providing adequate rehabilitation for individuals with neurological disorders [36].

Physical therapy (physical exercises and muscle strengthening) is well accepted as an important part of the interdisciplinary treatment of individuals with MMC, but there is no consensus or available trials on the frequency, intensity or what physiotherapeutic strategies should be used at different ages. However, specialists report that exercises are essential to enhancing mobility in children with MMC and avoiding the deterioration of motor function during growth [37,38]. A systematic review on physical training for individuals with spina bifida found a poorer physical fitness in this population compared to healthy peers and that aerobic and strength training seems to improve cardiorespiratory endurance and muscle strength. However, the authors concluded that further studies are needed to strengthen evidence-based recommendations [39].

Despite advances in medical and surgical management, neurological disorders continue to cause major disability burden for long periods of time. Advances in the understanding of recovery from CNS injuries and the development of new technologies have led researchers to explore novel interventions to promote the functional recovery of patients with neurological disorders [36].

In a study with a similar physiotherapeutic program as that described in the present protocol study, Aizawa et al. [4] investigated motor capacity and functional independence in children with MMC. The authors compared the effects of conventional physical therapy and a physiotherapeutic program based on reflex stimulation. Conventional physical therapy consisted of muscle strengthening and exercises involving changes of position (sitting, rolling, crawling, kneeling, semi-kneeling and standing). The authors found improvements in mobility and functioning in both treatment groups based on the scales of the Gross Motor Function Measure and PEDI.

To enable improvements in functional capacity and quality of life, Silva et al. [40] performed physical therapy combined with PBM in patients with spinal cord injuries. The physiotherapeutic protocol consisted of stretching and muscle strengthening exercises. The group submitted to combined treatment (physical therapy and PBM) demonstrated greater improvements in sensory and motor recovery compared to the group submitted to physical therapy alone. In another clinical trial, Silva et al. [14] provided evidence that PBM led to improvements in the motor response of individuals with spinal cord injuries, as demonstrated by differences in electromyographic signals before and after treatment. These results motivated the present treatment protocol for children with MMC.

The effects of PBM have been investigated in studies involving experimental spinal cord injury models. Wu et al. [41] demonstrated improvements in functional recovery and axonal regeneration following PBM at wavelengths of 808 and 700 nm administrated daily for 14 days. In another experimental model, Paula et al. [42] demonstrated a positive effect of PBM at a wavelength of 780 nm (administered at five points in the region of the injury) on the recovery of the spinal cord and, consequently, faster functional recovery. Veronez et al. [12] found an improvement in tactile sensitivity following transcutaneous PBM at a wavelength of 808 nm when administered with 1000 J/cm^2^ to a single point in the region of the injury.

With the PBM protocol in the studies by Silva et al. [14,20], individuals with spinal cord injuries received low-level laser irradiation over five points in the areas of the injury. The PBM parameters were 808 nm, aperture diameter of 0.18 cm, 25 J per point and an approximate application time of 17 minutes. Sessions were held three times per week for a total of 12 sessions. However, the study proposed herein will involve a LED device with a wavelength of 808 nm, aperture diameter of 1.9 cm and 25 J per point. Four points will be irradiated at the level of the injury with an approximate application time of 3 minutes.

In recent decades, PBM has been introduced as an innovative modality for stimulating neural activity and the central nervous system. This technique is based on the exposure of neural tissue to the low fluence of light at a wavelength ranging from red to infrared [43]. Both low-level laser and LED produce similar wavelengths. However, LED has planar arrays, which increase the beam area significantly, making it easier to treat large areas in a shorter time. Another important factor is that LED is a less expensive device compared to laser devices, making it more accessible [8].

### Strengths and weaknesses of the study

One of the strengths of this study is its design–a prospectively recorded, randomized, controlled, blind trial. The study will also involve allocation concealment and intention-to-treat analysis. The evaluator responsible for collecting the data will be blinded to the treatment group assignment. The physiotherapists responsible for the treatment have clinical experience and were trained by the main author of the study. Moreover, the study will be guided by the ICF-CY to ensure that the therapists conduct a broad assessment of the children’s activities and participation and determine the focus of the physical therapy intervention. The major limitation of the study is the lack of a control group with healthy children.

### Contribution to the physical therapy profession and to patients

There are few studies in the literature with intervention protocols for children with myelomeningocele. Therefore, this work is extremely important for physiotherapists and researchers. The possible discoveries with this work can guide healthcare providers in decision making regarding clinical care for this population.

The results of this study can contribute to a better understanding of the effectiveness of PBM on functioning and quality of life in children with myelomeningocele.

## Supporting information

S1 File Declaration of consent for participation in clinical research original (Guardian) (Original Portuguese). (DOCX)

S2 File Declaration of consent for participation in clinical research (Guardian) (English). (DOCX)

S3 File Declaration of consent for participation in clinical research original (Child) (Original Portuguese). (DOCX)

S4 File Declaration of consent for participation in clinical research (Child) (English). (DOCX)

S5 File SPIRIT. (DOCX)

S6 File Project_submitted to the ethics committee (Original Portuguese). (DOCX)

S7 File Project_submitted to the ethics committee (English). (DOCX)

S8 File OPINION ethics committe (Original Portuguese). (PDF)

S9 File OPINION ethics committe (English). (DOCX)

S10 File Database. (XLSX)
