# Supplementary material for: Effect of photobiomodulation combined with physical therapy on functional performance in children with myelomeningocele: A protocol randomized clinical blind study
Source: PLoS One. 2021 Oct 6;16(10):e0253963. doi: 10.1371/journal.pone.0253963 (PMC8494316; doi:10.1371/journal.pone.0253963)
Supplement: S1 File — (DOCX) [file pone.0253963.s001.docx]

**TCLE - Termo de Consentimento livre e esclarecido para Participação em Pesquisa Clínica**:

Nome do participante:____________________________________________________ Endereço:______________________________________________________________

Telefone para Contato:______________________ Cidade:_______________________ CEP:________ E-mail: ___________________________________________________

1.**Título do Trabalho Experimental:** Efeitos da fisioterapia associada com a fotobiomodulação no desempenho funcional em crianças com mielomeningocele- Estudo clínico, randomizado e cego

**2.Objetivo:** Avaliar se o tratamento fotobiomodulação (luz em baixa intensidade) associado a exercícios de fisioterapia melhora a força dos músculos da perna que estão fracos, e também melhorar a sensibilidade nas pernas, pois as crianças com mielomeningocele podem não sentir quando se machucam, ou quando são tocadas na perna.

**3.Justificativa:** a fotobiomodulação é um tratamento que usa uma luz em baixa intensidade. Essa luz sai de um aparelho e ao entrar em contato com a pele não provoca nenhum calor e nenhum incômodo. Ela vai atravessar a pele até chegar na “espinha” ou “medula espinal” da criança.

Foram realizados estudos em animais que sofreram lesão na sua coluna (lesão medular), que é semelhante a lesão que acontece na mielomeningocele. O tratamento nesses animais teve um efeito positivo na melhora da força muscular e sensibilidade. Além disso foi realizado um estudo em pacientes adultos que também sofreram lesão em sua coluna, afetando a medula e foram tratados com a luz em baixa intensidade e exercícios de fisioterapia, tiveram melhora na força muscular e sensibilidade. Queremos avaliar se o efeito dessa luz, em crianças com mielomeningocele associado a fisioterapia, pois pode ser uma alternativa de tratamento para a mielomeningocele, porém ainda não foi realizado nenhum trabalho com esse objetivo.

**4. Procedimentos da Fase Experimental:** O seu filho (a) está sendo convidado a participar desta pesquisa. A participação da criança poderá contribuir para a ampliação do conhecimento sobre a mielomeningocele, e o efeito doa luz em baixa intensidade associado a fisioterapia na força muscular e sensibilidade. Nós vamos realizar uma avaliação inicial, onde você irá nos contar a história da sua gravidez e parto e como foi realizado a cirurgia da mielomeningocele. Também vamos avaliar a força muscular da criança através da eletromiografia. A eletromiografia é um exame que basicamente vai avaliar a força das pernas da criança. Será colocado adesivos em alguns músculos da perna da crianças e vamos pedir para levantar e sentar em uma cadeira por 3 vezes, o tempo dessa avaliação será de aproximadamente 30 minutos, não se preocupe que seu filho não vai sentir dor. A criança também será avaliada através de monofilamentos, para saber se ela possui diminuição na sensibilidade das pernas. Os monofilamentos são 6 fios de nylon, coloridos e cada um tem um peso diferente. A criança vai fechar os olhos e vamos passar os fios na perna da criança e ela tem que responder se está sentindo. Essa avaliação será realizada através de uma brincadeira. O tempo dessa avaliação será de aproximadamente 15 minutos. E também não dói. Além disso a criança terá que cuspir em um tubinho, pois através da saliva nós vamos avaliar se houve produção de algumas substâncias que vão nos ajudar a entender o efeito dessa luz na força muscular e sensibilidade. Após a análise da saliva, esse material será descartado. Todas essas avaliações serão realizadas no início e final do tratamento e depois de 30 dias do término do tratamento.

Além disso, o Sr(a) terá que responder algumas perguntas de escalas de avaliação que avaliam o quanto seu filho(a) é independente nas atividades que ele realiza no dia-a-dia e também para que possamos avaliar a qualidade de vida do seu fiho(a).

Será realizado um sorteio para definir qual grupo experimental a criança fará parte. Pois um grupo vai realizar exercícios de fisioterapia e aplicação da luz, porém terá um grupo onde a criança vai realizar os exercícios de fisioterapia e o placebo da luz, ou seja, o aparelho não emitir a luz. Apesar disso fique tranquilo (a), pois após o término das sessões, se houver melhora na força e sensibilidade no grupo que realizou aplicação da luz e os exercícios, o grupo que realizou o placebo também vai realizar a aplicação real da luz, para que não fique em desvantagens.

A aplicação da luz em baixa intensidade será realizada em cima do nível da lesão, ou seja, na cicatriz cirúrgica, a criança ficará deitada de lado, e pode até ver um livrinho, ou ficar conversando. A aplicação será rápida no tempo de 5 minutos. A pesquisa será realizada 24 sessões 2 vezes na semana. Totalizando 12 semanas.

Se a criança faltar por 2 vezes consecutivas ou tiver 3 faltas não consecutivas deverá descontinuar a intervenção.

**5.Desconforto ou Riscos Esperados:** Pode ser desconfortável para a criança ficar deitada para aplicação da luz de baixa intensidade por 5 minutos. Lembrando que mesmo que mínimos, sempre existem riscos. Os dias de avaliação da eletromiografia podem ser desconfortáveis para a criança, pois o tempo de avaliação é de 30 minutos, e a criança poderá se sentir impaciente. Também pode ser desconfortável a coleta de saliva, pois a criança precisa cuspir no tubinho.

6. **Medidas protetivas aos riscos:** A criança será posicionada de forma confortável para aplicação da luz de baixa intensidade. Na coleta da saliva a criança irá cuspir em um tubinho novo e estéril, além disso os profissionais vão fazer uso de luvas e aventais. Todos os brinquedos utilizados para o desenvolvimento dos exercícios de fisioterapia serão devidamente limpos. O aparelho da luz também será coberto com plástico para evitar o contato direto com a pele.

**7. Benefícios da Pesquisa:** A criança irá realizar fisioterapia, os exercícios vão ser de acordo com o objetivo funcional e q queixa principal da criança/responsável.

**9. Retirada do Consentimento:** o participante e responsável tem a liberdade de retirar seu consentimento a qualquer momento e deixar de participar do estudo.

**10. Garantia do Sigilo:** O participante tem a garantia que receberá respostas a qualquer pergunta ou esclarecimento de quaisquer dúvidas quanto aos procedimentos, riscos, benefícios e outros assuntos relacionados com a pesquisa. Também os pesquisadores citados assumem o compromisso de proporcionar informação atualizada obtida durante o estudo, ainda que esta possa afetar a vontade do indivíduo em continuar participando.

**11. Formas de Ressarcimento das Despesas decorrentes da Participação na Pesquisa:**

Não será cobrada a sua participação na pesquisa, porém não serão ressarcidas despesas com eventuais deslocamentos.

**12. Local da Pesquisa:** A pesquisa será desenvolvida na UNINOVE campus VERGUEIRO, que fica localizado na rua Vergueiro, 235/249 - Liberdade, São Paulo - SP, Brasil.

**13. Comitê de Ética em Pesquisa (CEP)** é um colegiado interdisciplinar e independente, que deve existir nas instituições que realizam pesquisas envolvendo seres humanos no Brasil, criado para defender os interesses dos participantes de pesquisas em sua integridade e dignidade e para contribuir no desenvolvimento das pesquisas dentro dos padrões éticos (Normas e Diretrizes Regulamentadoras da Pesquisa envolvendo Seres Humanos – Res. CNS nº 466/12 e Res. CNS 510/2016). O Comitê de Ética é responsável pela avaliação e acompanhamento dos protocolos de pesquisa no que corresponde aos aspectos éticos. Endereço do Comitê de Ética da Uninove: Rua. Vergueiro nº 235/249 – 12º andar - Liberdade – São Paulo – SP CEP. 01504-001 Fone: 3385-9010 comitedeetica@uninove.br Horários de atendimento do Comitê de Ética: segunda-feira a sexta-feira – Das 11h30 às 13h00 e Das 15h30 às 19h00 14.

A qualquer momento você poderá entrar em contato com os pesquisados nos telefones: Prof. Dr. Sandra Kalil Bussadori - (011) 983817453, Aluna Tamiris da Silva - (011) 976764625.

15. Eventuais intercorrências que vierem a surgir no decorrer da pesquisa poderão ser discutidas pelos meios próprios.

São Paulo, de 20

**16.Consentimento Pós-Informação:**

Eu, ________________________________________________, após leitura e compreensão deste termo de informação e consentimento, entendo que minha participação é voluntária, e que posso sair a qualquer momento do estudo, sem prejuízo algum. Confirmo que recebi uma via deste termo de consentimento, e autorizo a realização do trabalho de pesquisa e a divulgação dos dados obtidos somente neste estudo no meio científico.

_______________________________

Assinatura do Participante

(Todas as folhas devem ser rubricadas pelo participante da pesquisa)

**17.** Eu, ________________________________________ (Pesquisador do responsável desta

pesquisa), certifico que:

a) Esta pesquisa só terá início após a aprovação do(s) referido(s) Comitê(s) de Ética em Pesquisa o qual o projeto foi submetido.

b) Considerando que a ética em pesquisa implica o respeito pela dignidade humana e a proteção devida aos participantes das pesquisas científicas envolvendo seres humanos;

c) Este estudo tem mérito científico e a equipe de profissionais devidamente citados neste termo é treinada, capacitada e competente para executar os procedimentos descritos neste termo;

_______________________________________________

(COLOCAR O NOME COMPLETO DO PESQUISADOR RESPONSÁVEL)

Assinatura do Pesquisador Responsável
