# Supplementary material for: Effect of photobiomodulation combined with physical therapy on functional performance in children with myelomeningocele: A protocol randomized clinical blind study
Source: PLoS One. 2021 Oct 6;16(10):e0253963. doi: 10.1371/journal.pone.0253963 (PMC8494316; doi:10.1371/journal.pone.0253963)
Supplement: S2 File — (DOCX) [file pone.0253963.s002.docx]

**Informed Consent Form for Participation in Clinical Research:**

Name of participant: ____________________________________________________

Address: ______________________________________________________________

Telephone number: ______________________

City: _______________________ Zip code: ________

E-mail: ___________________________________________________

**1.Title of Experimental Work:** Effects of physical therapy associated with photobiomodulation on functional performance in children with myelomeningocele- Clinical, randomized and blind study

**2.Objective:** To assess whether photobiomodulation treatment (low-intensity light) associated with physiotherapy exercises improves the strength of weak leg muscles, and also improves leg sensitivity, as children with myelomeningocele may not feel when they get hurt , or when they are touched on the leg.

**3. Justification:** photobiomodulation is a treatment that uses low intensity light. This light comes out of a device and when it comes in contact with the skin it does not cause any heat and no discomfort. It will pass through the skin until it reaches the child's "spine" or "spinal cord".

Studies have been carried out on animals that have suffered an injury to their spine (spinal cord injury), which is similar to the injury that occurs in myelomeningocele. Treatment in these animals had a positive effect on improving muscle strength and sensitivity. In addition, a study was carried out on adult patients who also suffered spinal injuries, affecting the spinal cord and were treated with low intensity light and physiotherapy exercises, who had improved muscle strength and sensitivity. We want to evaluate whether the effect of this light, in children with myelomeningocele associated with physical therapy, as it can be an alternative treatment for myelomeningocele, however, no work has yet been carried out with this objective.

**4. Experimental Phase Procedures:** Your child is being invited to participate in this research. The child's participation may contribute to the expansion of knowledge about myelomeningocele, and the effect of donating light at low intensity associated with physiotherapy on muscle strength and sensitivity. We will carry out an initial assessment, where you will tell us the story of your pregnancy and delivery and how myelomeningocele surgery was performed. We will also assess the child's muscle strength through electromyography. Electromyography is an exam that will basically assess the strength of the child's legs. Stickers will be placed on some of the children's leg muscles and we will ask you to get up and sit on a chair 3 times, the time for this assessment will be approximately 30 minutes, don't worry that your child will not feel pain. The child will also be evaluated through monofilaments, to see if he has decreased sensitivity in the legs. The monofilaments are 6 nylon threads, colored and each one has a different weight. The child will close his eyes and we will put the threads on the child's leg and he has to answer if he is feeling. This evaluation will be performed through a game. This assessment will take approximately 15 minutes. It also doesn't hurt. In addition, the child will have to spit in a tube, because through the saliva we will assess whether there has been the production of some substances that will help us understand the effect of this light on muscle strength and sensitivity. After saliva analysis, this material will be discarded. All of these assessments will be carried out at the beginning and end of treatment and after 30 days after the end of treatment.

In addition, you will have to answer some questions of assessment scales that assess how independent your child is in the activities that he performs on a daily basis and also so that we can assess the quality of life of the child. your child.

A drawing will be carried out to define which experimental group the child will be part of. For a group will perform physiotherapy exercises and apply light, but there will be a group where the child will perform physiotherapy exercises and the placebo of light, that is, the device will not emit light. In spite of this, rest assured that, after the end of the sessions, if there is an improvement in strength and sensitivity in the group that applied the light and the exercises, the group that performed the placebo will also perform the actual application of the light, don't be at a disadvantage.

The application of light at low intensity will be carried out above the level of the lesion, that is, in the surgical scar, the child will lie on its side, and may even see a little book, or be talking. The application will be quick in 5 minutes. The survey will be conducted 24 sessions 2 times a week. Totaling 12 weeks.

If the child is absent for 2 consecutive times or has 3 non-consecutive absences, the intervention must be discontinued.

**5. Discomfort or Expected Risks:** It can be uncomfortable for the child to lie down to apply low intensity light for 5 minutes. Remembering that even if they are minimal, there are always risks. The electromyography evaluation days can be uncomfortable for the child, as the evaluation time is 30 minutes, and the child may feel impatient. It can also be uncomfortable to collect saliva, as the child needs to spit in the tube.

**6. Protective measures against risks:** The child will be positioned comfortably for the application of low intensity light. In the collection of saliva the child will spit in a new and sterile tube, in addition the professionals will use gloves and aprons. All toys used for the development of physiotherapy exercises will be properly cleaned. The light fixture will also be covered with plastic to prevent direct contact with the skin.

**7. Research Benefits:** The child will undergo physical therapy, the exercises will be in accordance with the functional objective and q main complaint of the child / guardian.

**9. Withdrawal of Consent:** the participant and guardian is free to withdraw their consent at any time and stop participating in the study.

**10. Secrecy Guarantee:** The participant has the guarantee that he will receive answers to any question or clarification of any doubts regarding the procedures, risks, benefits and other matters related to the research. The aforementioned researchers are also committed to providing updated information obtained during the study, even though this may affect the individual's willingness to continue participating.

**11. Forms of Reimbursement of Expenses resulting from Participation in the Research:** You will not be charged for your participation in the survey, but expenses with any travel will not be reimbursed.

**12. Research Location:** The research will be carried out at UNINOVE campus VERGUEIRO, which is located at Rua Vergueiro, 235/249 - Liberdade, São Paulo - SP, Brazil.

**13. Research Ethics Committee (CEP)** is an interdisciplinary and independent collegiate body, which must exist in institutions that carry out research involving human beings in Brazil, created to defend the interests of research participants in their integrity and dignity and to contribute to development of research within ethical standards (Norms and Regulatory Guidelines for Research involving Human Beings - Res. CNS nº 466/12 and Res. CNS 510/2016). The Ethics Committee is responsible for the evaluation and monitoring of research protocols in terms of ethical aspects. Uninove Ethics Committee address: Rua. Vergueiro nº 235/249 - 12th floor - Liberdade - São Paulo - SP CEP. 01504-001 Phone: 3385-9010 comitedeetica@uninove.br Opening hours of the Ethics Committee: Monday to Friday - From 11:30 am to 1:00 pm and from 3:30 pm to 7:00 pm 14.

At any time you can contact the respondents on the phones: Dr. Sandra Kalil Bussadori - (011) 983817453, Tamiris da Silva - (011) 976764625.

15. Eventual complications that may arise in the course of the research may be discussed by the proper means.

São Paulo, de 20

**16. Post-Information Consent:**

I, ________________________________________________, after reading and understanding this term of information and consent, I understand that my participation is voluntary, and that I can leave the study at any time, without prejudice. I confirm that I received a copy of this consent form, and authorize the research work and the dissemination of data obtained only in this study in the scientific community.

___________________________

Participant's Signature

(All pages must be initialed by the research participant)

17. I, ________________________________________ (Researcher in charge of this

I certify that:

a) This research will only start after the approval of the referred Research Ethics Committee (s) to which the project was submitted.

b) Considering that research ethics implies respect for human dignity and protection due to participants in scientific research involving human beings;

c) This study has scientific merit and the team of professionals duly mentioned in this term is trained, qualified and competent to perform the procedures described in this term;

_______________________________________________

(PUT THE FULL NAME OF THE RESPONSIBLE RESEARCHER)

Signature of the Responsible Researcher
