# Supplementary material for: Effect of photobiomodulation combined with physical therapy on functional performance in children with myelomeningocele: A protocol randomized clinical blind study
Source: PLoS One. 2021 Oct 6;16(10):e0253963. doi: 10.1371/journal.pone.0253963 (PMC8494316; doi:10.1371/journal.pone.0253963)
Supplement: S3 File — (DOCX) [file pone.0253963.s003.docx]

.

**TERMO DE ASSENTIMENTO**

**Você está sendo convidado (a) a participar da pesquisa:**

**Efeitos da fisioterapia associada com a fotobiomodulação no desempenho funcional em crianças com mielomeningocele- Estudo clínico, randomizado e cego, CONTROLADO E CEGO**

**O adulto que é responsável por você nos contou que quando você nasceu, tinha uma bolsinha nas suas costas e o médico retirou.**

**Por conta dessa bolsinha você ficou com fraqueza em suas perninhas e não as sente muito.**


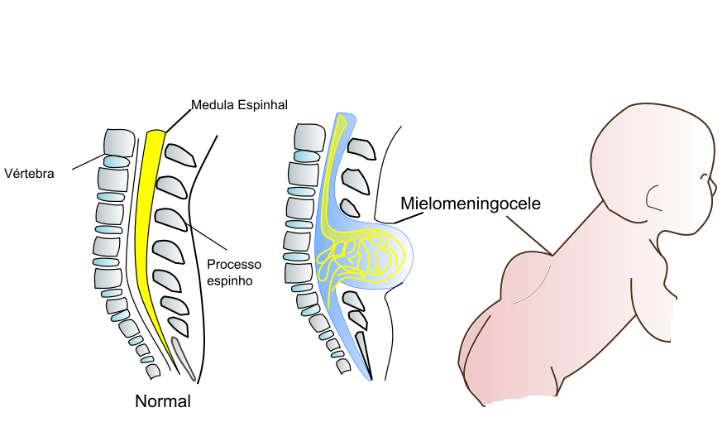


**QUEREMOS APLICAR UMA LUZ EM VOCÊ, PARA SABER SE SUAS PERNINHAS VÃO MELHORAR.**

**NÃO SE PREOCUPE NÃO VAI DOER.**


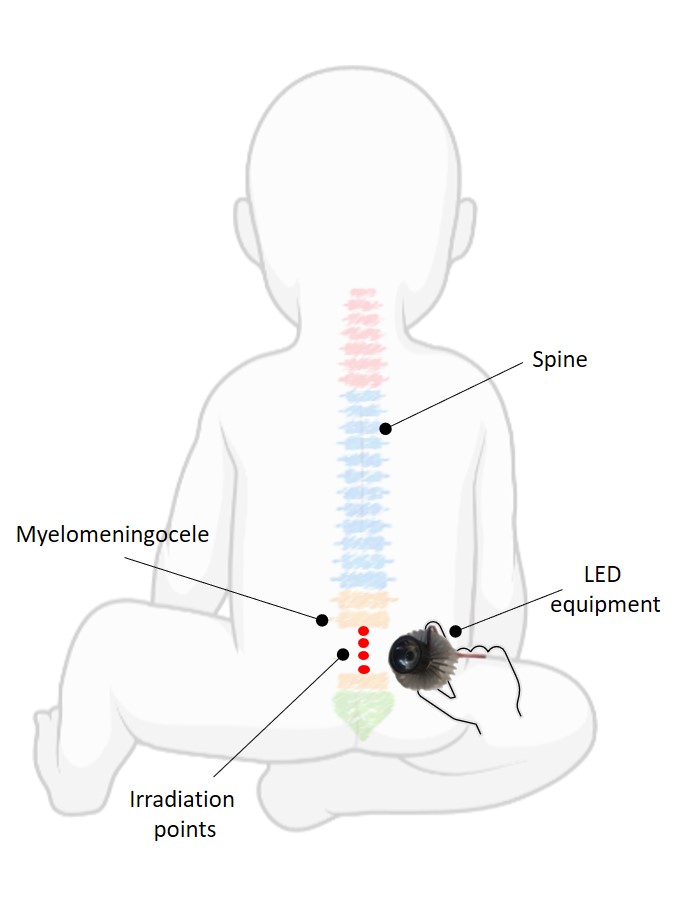


**Primeiro iremos avaliar a força das suas perninhas.**

**Não se preocupe não vai doer**

**Vou colocar alguns adesivos na sua perna**

**E vamos pedir para que você levante e sente novamente na cadeira**

**Além disso tudo, iremos brincar, rir e se divertir bastante durante os exercícios DE FISIOTERAPIA que faremos juntos.**

**Seus pais deixaram você brincar com a gente, mas você não é obrigado e se não quiser não tem problema, ninguém irá ficar bravo com você. Deixe o seu dedinho:**

**SIM, QUERO PARTICIPAR.**

**NÃO, NÃO QUERO PARTICIPAR.**

**SIM, ACEITO PARTICIPAR.**

CONTATO: **(11) 9 76764625** Tamiris Silva

**(11) 9 8381-7453** Dra. Sandra Kalil Bussadori

**E-mail:** [**tamiris.slv@hotmail.com**](mailto:tamiris.slv@hotmail.com)
