# Supplementary material for: Effect of photobiomodulation combined with physical therapy on functional performance in children with myelomeningocele: A protocol randomized clinical blind study
Source: PLoS One. 2021 Oct 6;16(10):e0253963. doi: 10.1371/journal.pone.0253963 (PMC8494316; doi:10.1371/journal.pone.0253963)
Supplement: S4 File — (DOCX) [file pone.0253963.s004.docx]

.

**TERMS OF ASSENT**

**You are being invited to participate in the survey:**

**Effects of physical therapy associated with photobiomodulation on functional performance in children with myelomeningocele- Clinical study, randomized and blind, CONTROLLED AND BLIND**

**The adult who is responsible for you told us that when you were born, you had a little bag on your back and the doctor removed it.**

**Because of that little bag you got weak in your little legs and don't feel them very much.**


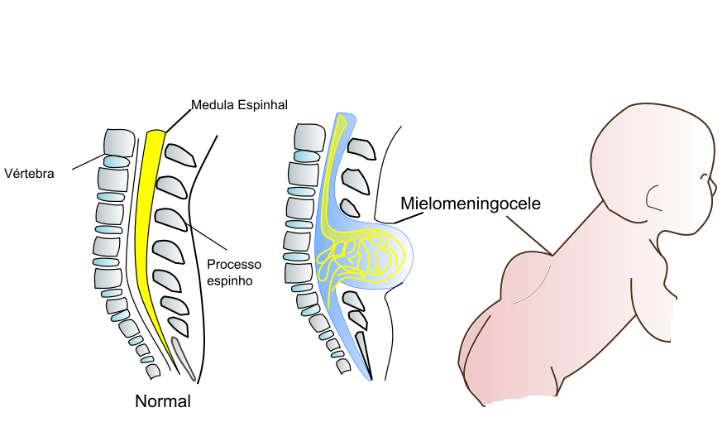


**WE WANT TO APPLY A LIGHT ON YOU, TO KNOW IF YOUR LEGS WILL IMPROVE.**

**DON'T WORRY IT WILL NOT HURT.**


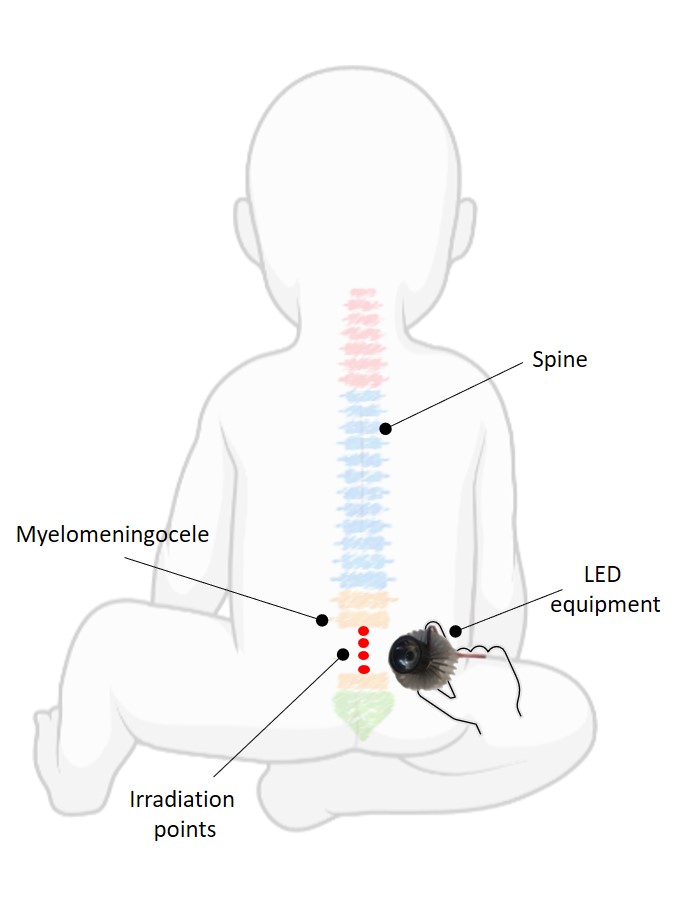


**First we will assess the strength of your legs.**

**I'll put some stickers on your leg**

**And let's ask you to get up and sit back in the chair**

**don't worry it won't hurt**

**In addition, we will play, laugh and have a lot of fun during the PHYSIOTHERAPY exercises that we will do together.**

**Your parents let you play with us, but you don't have to and if you don't have a problem, no one will be mad at you. Leave your little finger:**

**Deixe o seu dedinho:**

**YES, I DO**

**I don't want to participate**

**SIM, ACEITO PARTICIPAR.**

CONTAT: **(11) 9 76764625** Tamiris Silva

**(11) 9 8381-7453** Dra. Sandra Kalil Bussadori

**E-mail:** [**tamiris.slv@hotmail.com**](mailto:tamiris.slv@hotmail.com)
