# Supplementary material for: Effect of photobiomodulation combined with physical therapy on functional performance in children with myelomeningocele: A protocol randomized clinical blind study
Source: PLoS One. 2021 Oct 6;16(10):e0253963. doi: 10.1371/journal.pone.0253963 (PMC8494316; doi:10.1371/journal.pone.0253963)
Supplement: S6 File — (DOCX) [file pone.0253963.s006.docx]

**Efeitos da fisioterapia associada com a fotobiomodulação no desempenho funcional em crianças com mielomeningocele- Estudo clínico, randomizado e cego**

**Aluna de doutorado:** Tamiris da Silva

**Orientadora:** Profª Drª. Sandra Kalil Bussadori

**Instituição:** Universidade Nove de Julho

**Programa de Pós-graduação em Ciências da**

**Reabilitação**

Resumo

A mielomeningocele é um tipo grave de espinha bífida, resultante do fechamento inadequado do tubo neural. Essa condição afeta drasticamente as estruturas da medula espinhal, como resultado, a medula espinhal, as raízes nervosas e as meninges são expostas durante a gravidez, resultando em deficiências. A combinação dessas deficiências resulta em uma diminuição geral da mobilidade e da participação funcional. Há poucas evidências sobre intervenções fisioterapêuticas nessa população. No entanto, sabe-se que a Classificação Internacional de Funcionalidade, Incapacidade e Saúde para Crianças e Jovens é uma ferramenta útil para auxiliar os terapeutas na análise de problemas e na reflexão sobre o foco da intervenção. A literatura atual demonstra que recursos como a luz de baixa intensidade, também conhecida como fotobiomodulação como meio terapêutico, podem ser meios auxiliares na reabilitação de condições neurológicas, pois estudos mostram que a fotobiomodução promove recuperação sensorial e motora no modelo animal de lesão medular. E um ensaio clínico mostrou que, após o tratamento com fotobiomodulação combinado com a fotobiomodulação, indivíduos com lesão medular melhoraram a função motora e sensorial. Portanto, o objetivo do estudo é avaliar os efeitos da fisioterapia associada à fotobiomodulação no desempenho funcional de crianças com mielomeningocele lombar baixa e sacral. Materiais e métodos: serão recrutados na Clínica Integrada de Saúde da Universidade Nove de Julho. Aqueles que atenderem aos critérios de inclusão serão randomizados para dois grupos usando um site de randomização (randomization.com). O grupo 1 será submetido a PBM ativo e exercícios fisioterapêuticos. O grupo 2 será submetido a simulações de PBM e exercícios fisioterapêuticos. A irradiação será realizada com um LED com comprimento de onda de 850 nm, energia por ponto de 25 J, 50 segundos por ponto e potência de 200 mW. no grupo placebo, o dispositivo não emitirá luz. Os objetivos dos exercícios de fisioterapia serão realizados de acordo com as metas e objetivos da criança / responsáveis. o protocolo de tratamento será duas vezes na semana por 24 sessões. as avaliações serão executadas antes do tratamento, na última sessão de tratamento e 30 dias após o tratamento. A atividade muscular será avaliada usando uma eletromiografia portátil (BTS Engineering) e, como medida de funcionalidade, a tarefa de sentar e levantar será realizada. Os eletrodos serão posicionados sobre os músculos: gastrocnêmio lateral, tibial anterior e reto femoral. Para avaliar a independência funcional das crianças, será utilizado o Inventário de Avaliação Pediátrica da Incapacidade. A qualidade de vida será avaliada pelo Questionário de Saúde da Criança - Formulário 50 dos pais. A avaliação sensorial será realizada com o kit Semmes-Weinstein (Smiles®). A expressão proteica do BDNF será quantificada a partir de amostras de saliva usando a técnica ELISA. Os dados serão analisados ​​com o auxílio do GraphPad PRISM versão 7.0.

**Key Words:** Myelomeningocele, Physical Therapy Modalities, Physical Functional Performance, photobiomodulation, Electromyography

**Introdução**

A mielomeningocele (MMC) é um tipo grave de espinha bífida, resultante do fechamento inadequado do tubo neural.(1) A etiologia multifatorial da MMC está relacionada a fatores ambientais e maternos. A incidência global da doença é de um em cada 1000 nascidos vivos.(2) Essa condição afeta drasticamente as estruturas da medula espinhal, visto que as vértebras ao nível da lesão não têm processo espinhoso sendo portanto incompletas dorsalmente, este fator faz com que a medula espinhal, raízes nervosas e meninges sejam expostas durante a gestação resultando em deficiências incluindo paraplegia, deformidades esqueléticas, fraqueza muscular, perda de sensação, coordenação deficiente, equilíbrio diminuído, hidrocefalia, malformação de Arnold Chiari, e disfunções fecal, urinária e sexual. A combinação dessas deficiências resulta em uma diminuição geral da mobilidade e participação funcional. (3)

A patogênese da MMC não está totalmente elucidada, porém evidências crescentes indicam que os danos na medula espinhal associados a MMC ocorram devido a causa primária a qual está relacionada ao desenvolvimento anormal da medula espinhal e a causa secundária que ocorre devido as lesões traumáticas e químicas subsequentes da exposição da medula e estão associados à perda da função neurológica em fetos com MMC. (4,5) Após uma lesão no sistema nervoso central (SNC), não há recuperação da maioria dos axônios, em virtude de falhas regenerativas que ocorrem após danos no SNC, o que geralmente induz a incapacidades permanentes. Estas sequelas permanentes ocorrem porque os astrócitos formam cicatrizes com objetivo de restringir a inflamação e preservar o tecido neural, sendo este um processo essencial para a cicatrização, por outro lado, essas cicatrizes crônicas são prejudiciais pois impedem continuamente a regeneração do axônio.(6)

Sendo assim, as estratégias de tratamento para essa condição possuem o objetivo de minimizar a extensão da lesão reduzindo as possíveis sequelas. O tratamento clássico para a MMC consiste no fechamento cirúrgico do defeito da MMC ainda na fase intrauterina ou logo após o nascimento. Estudos comparativos entre o reparo pré-natal versus o pós-natal demonstraram que em ambos os tratamentos o comprometimento funcional abaixo do nível da lesão permanece incompleta.(4)

O comprometimento funcional foi classificado por Hoffer et al.(7) em níveis funcionais de acordo com o comprometimento neurológico: torácico, lombar alto, lombar baixo e sacral. O prognóstico de deambulação e os objetivos a serem alcançados na reabilitação dependem não somente do nível neurológico, mas também da presença ou não de deformidades ortopédicas, obesidade, rebaixamento do cognitivo e condições sócio-econômicas da família.(8)

Em relação as intervenções fisioterapêuticas, tem-se o conhecimento de que se concentram em objetivos de otimizar a mobilidade e maximizar a independência e participação, o que pode ser facilitado pelo fortalecimento muscular, posicionamento adaptativo e / ou melhor controle postural. No entanto, as evidências sobre a eficácia dos exercícios de fisioterapia em crianças com MMC são limitadas.(9)

Sabe-se que a Classificação Internacional de Funcionalidade, Incapacidade e Saúde para Crianças e Jovens (CIF-CY) é uma ferramenta útil para auxiliar os terapeutas quanto a análise de problemas e no pensamento sobre o foco da intervenção. Durante muito tempo, as intervenções concentraram-se nas deficiências funcionais e no nível da estrutura do corpo, ou seja, nas habilidades da criança. Mais recentemente, uma mudança foi observada na literatura em direção a uma abordagem na qual as intervenções se concentram nos fatores ambientais, ou seja, na alteração de restrições em uma tarefa ou no ambiente da criança, enfatizando desta forma mais eficiente de concluir uma atividade. (10,11)

As evidências em prática de fisioterapia para indivíduos de todas as idades com distúrbios neurológicos, foca em 5 Ps: prevenção, predição, participação, personalizado e plasticidade. Sendo assim, os fisioterapeutas neurológicos devem realizar o atendimento com foco em prevenir as incapacidades do paciente, prever a resposta ideal de uma intervenção, através de medidas de resultados do sistema do movimento. Além disso, é de suma importância que o objetivo da reabilitação seja que as pessoas com deficiências neurológicas sejam totalmente incluídas e participem de atividades da vida que são importantes para elas e que seu atendimento seja personalizado. Todas essas medidas facilitam o processo de plasticidade positiva. (12,13)

A neuroplasticidade refere-se à tendência dos circuitos neurais de sofrerem alterações fisiológicas e/ou estruturais em resposta a mudanças nos padrões provocadas por lesões e/ou influências ambientais, ou seja, ocorre aumento da angiogênese e sinapses Embora a neuroplasticidade seja mais comumente associada ao córtex cerebral, todas as partes do sistema nervoso, incluindo a medula espinhal, demonstram plasticidade, a exemplo dos mecanismos sinápticos de aprendizado e memória, poda dendrítica, brotação colateral e regeneração axonal.(14) Outro fator que influencia e regula a neuroplasticidade é o fator neutrófico de crescimento neural (BDNF), as funções mais importantes do BDNF incluem, regulação da sinaptogênese, neuroproteção e aumento da arborização dendrítica, além disso o BDNF influencia os aspectos funcionais e estruturais da transmissão sináptica. A reabilitação induz a neuroplasticidade que pode ser evidenciada através da melhora do desempenho funcional e aumento de BDNF. (15)

A literatura atual demonstra que recursos que se utilizem da luz como meio terapêutico podem ser utilizados como auxiliares na reabilitação de condições neurológicas, a exemplo acidente vascular cerebral, doenças neurodegenerativas e lesões da medula espinhal. A Fotobiomodulação (PBM) ocorre a partir da aplicação de luz de baixa intensidade (luz vermelha e infravermelha), a exemplo do laser de baixa intensidade e diodo emissor de luz (LED) em tecidos biológicos. A eficácia terapêutica da Fototerapia é baseada na absorção de fótons por fotorreceptores ou cromóforos. (16,17)

 Estudos no modelo experimental de lesão medular, os autores demonstraram que tanto o comprimento de onda vermelho e o infravermelho têm o potencial de serem meios eficazes e não invasivos, de terapia, promovendo o brotamento axonal, aumento na concentração de células gliais e de conexões nervosas, além de melhora funcional e sensitiva. (18,19) Os achados de um ensaio clínico envolvendo indivíduos com uma o diagnóstico de lesão medular demonstra que a PBM exerceu efeitos positivos na função motora, principalmente durante a contração isotônica dos músculos estimulados avaliados por eletromiografia (EMG).(20) Além disso, Silva et al.(21) demonstraram que após 12 sessões de fotobiomodulação associado a fisioterapia em pacientes com lesão medular, houve recuperação na percepção sensorial e força muscular. Portanto, a FBM pode ser um tratamento promissor associado aos exercícios de fisioterapia com MMC.

**Justificativa**

O presente estudo propõe realizar um ensaio clínico controlado para avaliar a efetividade da fisioterapia associada a FBM como tratamento complementar na melhora sensorial e motora em indivíduos com MMC. A FBM é um tratamento não invasivo, e um procedimento rápido e pode ser abordagem promissora no tratamento, porém a literatura se mostrou escassa de estudos sobre o assunto. A MMC assim como na lesão medular, é um trauma severo do sistema nervoso e ainda não existem terapias restauradoras eficazes, no entanto, a FBM apresenta efeitos biomoduladores no tecido do sistema nervoso central e periférico.

Caso existam resultados positivos, é possível propor uma nova forma de tratamento para a resposta sensório-motora nesses indivíduos.

**Objetivos**

Avaliar o desempenho funcional de crianças com mielomeningocele após exercícios de fisioterapia associada a fotobiomodulação.

**Objetivos Secundários**

-Avaliar a sensibilidades superficial com os monofilamentos Semmes-Weinstein

- Avaliar a independência funcional através da escala PEDI

- Avaliar a qualidade de vida através Questionário de Saúde das Crianças – Relatório dos Pais 50

- Avaliar a expressão proteica dos níveis de BDNF nas amostras de saliva por ELISA

**Materiais e Métodos**

Este estudo será um ensaio clínico, controlado e randomizado que será desenvolvido segundo o fluxograma apresentado na Figura 1.

Este estudo seguirá as normas regulamentadoras de pesquisa em seres humanos com submissão e aprovação do Comitê de Ética (CNS nº 466/12 e Res. CNS 510/2016) em pesquisa da Universidade Nove de Julho.

Após a aprovação do comitê de ética, será realizado convite para participar da pesquisa será realizado após o levantamento dos pacientes que realizam fisioterapia ou se encontram na lista de espera do ambulatório da UNINOVE. Os participantes serão convidados a realizar a triagem, na qual serão aplicadas ficha de avaliação inicial (anexo 1). Os participantes ou responsáveis assinarão o Termo de Consentimento Livre e Esclarecido (TCLE) (Anexo 2) e as crianças de 5-14 anos irão assinar o termo de assentimento (Anexo 3). Além disso, as crianças que não souberem ler, os pesquisadores, vão ler, explicar através das imagens todo procedimento que será realizado, e no final se a crianças concordar, os pesquisadores irão colocar tinta no dedo da crianças para que ela assine se quer ou não participar da pesquisa.

Recrutamento

incluídos

Excluídos

Grupo 1

Exercícios de fisioterapia

+

Fotobiomodulação ativa

Grupo 2

Exerícios de fisioterapia

+

Placebo da Fotobiomodulação

Avaliações

T0= pré-intervenção

T1= após 12 sessões de fisioterapia PBM + pós-intervenção

T2= 30 dias após a conclusão da intervenção (acompanhamento)

Figura 1. Desenho do estudo.

**Participantes**

A elegibilidade para participação no estudo dependerá dos seguintes critérios:

**Critérios de inclusão:** idade de 5 a 14 anos; diagnóstico de mielomeningocele no nível lombar e sacral inferior; Consiga Realizador ou Movimento de Sentar e Levantar com Apoio.

**Critérios de exclusão:** comprometimento cognitivo que compromete a capacidade de se comunicar e responder às perguntas que serão colocadas; alergia ao látex; manifestações secundárias ao MMC, como pé torto congênito; escoliose neuromuscular; subluxação ou luxação do quadril e joelho; outra doença do sistema nervoso central.

**Randomização**

Os participantes serão randomizados em dois grupos usando um site de randomização (randomization.com). O grupo 1 será submetido a Fotobiomodulação ativo e exercícios fisioterapêuticos. O grupo 2 será submetido a simulações de fotobiomodulação e exercícios fisioterapêuticos.

**Cegamento**

- Os participantes não saberão se qual grupo estão participando, ou seja, grupo de tratamento ou placebo com a fotobiomodulação.
- O avaliador e quem irá realizar os exercícios não saberá a qual grupo o participante está participando em relação a fotobiomodulação.
- Um terapeuta que não participará das avaliações e sessões da fisioterapia irá realizar a aplicação da fotobiomodulação.
- Os microtubos com as amostras de saliva será marcado com números correspondentes a cada participantes, assim o avaliador não saberá a qual grupo a amostra corresponde. Um terapeuta será responsável pelas escalas de avaliação e eletromiografia.

**Intervenções**

**Protocolo da fotobiomodulação**

Para a irradiação os indivíduos serão posicionados de forma confortável em decúbito lateral na maca. Será irradiado apenas 4 pontos, em cima do nível da lesão. O nível da lesão será localizado através do exame de imagem, que será solicitado para o responsável trazer no dia da avaliação. Após determinar o nível da lesão será realizada palpação dos processos transversos das vértebras.

Será utilizado o mesmo dispositivo de LED para ambos os grupos, entretanto, no grupo placebo o dispositivo de laser irá emitir som que irá delinear o início e o final da terapia, porém não emitirá luz. Serão realizadas doze sessões, duas vezes na semana utilizando os parâmetros descritos na Tabela 1.

Será realizado vinte e quatro sessões, duas vezes na semana.

| **Parâmetros** | **LED infravermelho** |
| --- | --- |
| Comprimento de onda [nm] | 850 |
| Modo de operação | Contínuo |
| Diâmetro [cm] | 0,4 |
| Área [cm^2^] | 0,126 |
| Irradiância [W/cm^2^] | 796 |
| Duração da exposição [s] | 50 (por ponto) |
| Exposição radiante [J/cm^2^] | 200 |
| Energia radiante [J] | 25 (por ponto) |
| Número de pontos irradiados | 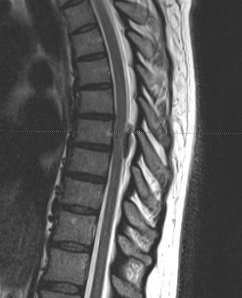4 |
| Técnica de aplicação | Contato |
| Número e a frequência das sessões  de tratamento | 2 vezes por semana  24 sessões |

**Fisioterapia**

Os exercícios de fisioterapia serão individualizados e personalizados para cada criança. Levará em consideração reclamações e / ou atrasos no motor decorrentes da lesão. Serão realizados exercícios em circuito envolvendo exercícios de fortalecimento muscular, estimulação sensorial e equilíbrio. Todos os exercícios serão associados a atividades recreativas, como jogar bola, pescar. Os participantes também podem se envolver na história de princesas e super-heróis que lhes permitem realizar tarefas relacionadas aos objetivos.

A fisioterapia será duas vezes por semana por 45-60 minutos após a aplicação do LED por 24 sessões

**Critérios para descontinuar a intervenção**

Faltar por 2 vezes consecutivas ou ter 3 faltas não consecutivas deverá descontinuar a intervenção.

**Aderência**

Todas as intervenções são realizadas de forma individual. Para garantir a adesão ao protocolo predefinido, os fisioterapeutas serão instruídos pelo investigador principal sobre como fornecer as intervenções, bem como receber orientações dos exercícios. Os terapeutas registrarão como foi o treinamento em termos de tipo e execução de exercícios, nível de intensidade e repetições. O fisioterapeuta responsável pela intervenção na clínica de fisioterapia participante registra sintomas de possíveis eventos adversos relacionados ao tratamento. Qualquer evento adverso é então relatado ao investigador principal, que então se reporta ao comitê de ética regional.

**Avaliações**

As avaliações serão realizadas antes da terapia (pré-intervenção-T0), após 12 sessões de fisioterapia PBM + (pós-intervenção) (T1) e 30 dias após a conclusão da intervenção (acompanhamento) (T2).

**Eletromiografia de superfície**

A atividade muscular será avaliada através de um eletromiográfo portátil (BTS Engineering) sincronizado ao sistema BST EMG analizer. Como medida de funcionalidade será realizado a tarefa sit-to-stand. (22,23)

Os eletrodos serão posicionados nos músculos: gastrocnêmio lateral (GL), tibial anterior (TA) e reto femoral (RF).

O preparo da pele e a colocação dos eletrodos será de acordo com as diretrizes de avaliação não invasiva (SENIAN).(24) Os eletrodos serão posicionados com a criança em decúbito ventral (região de GL) e decúbito dorsal (região de TA e RF)

Para a avaliação do sit-to-stand o participante será posicionado em uma cadeira com 90º de flexão de quadril, joelho, tornozelo e pés apoiados.

Será realizado uma avaliação com a criança sentada em repouso. Após será solicitado que a criança se levante e permaneça em posição de ortostatismo por 10 segundos. Após será solicitado que a criança se sente. Será realizado a repetição da tarefa sit-to-stand 3 vezes com intervalo de 5 minutos.

As crianças vão realizar o movimento na velocidade que costumam adotar em sua rotina diária.

O tempo de coleta será de aproximadamente 30 minutos, sempre respeitando um intervalo de descanso entre cada coleta, para evitar os efeitos da fadiga.

**Avaliação sensorial**

A avaliação sensorial será realizada com o kit Semmes-Weinstein. O kit contém um conjunto de seis monofilamentos de nylon (estesiometria) do mesmo comprimento, que exercem força sobre a área específica testada. Cada monofilamento é representado por uma cor e diâmetro: Verde (0,05 g), azul (0,2 g), violeta (2 g), vermelho (4 g), laranja (10 g) e vermelho magenta (300 g) 16. O teste será realizado nos dermátomos (Os escores variaram de sete (monofilamento verde) a 1 (monofilamento vermelho magenta).^18^

Será avaliado os seguintes dermátomos: L1, L2, L4, L5 ,S1, S2.

O tempo de avaliação será de aproximadamente 15 minutos.

**Participação e Medida Ambiental**

A mudança na participação será avaliada usando a Medida de Participação e Meio Ambiente para Crianças e Jovens (PEM-CY). O PEM-CY é um instrumento de relatório dos pais que examina a participação e os fatores ambientais que afetam a participação das crianças em três ambientes: casa, escola e comunidade. Os pais serão solicitados a avaliar o envolvimento de seus filhos em 25 atividades nos três ambientes. Para cada atividade, o pai perguntará com que frequência seu filho participou de uma ou mais atividades desse tipo e a questão será avaliada em uma escala de 8 pontos, de nunca a diária. Além disso, eles serão solicitados a avaliar o envolvimento de seus filhos em cada uma das atividades usando uma escala de 5 pontos, de minimamente envolvidos a muito envolvidos, e se eles desejam que a participação de seus filhos mude neste tipo de atividade. (26)

**Habilidades funcionais**

Para avaliar a independência funcional das crianças será utilizado o Pediatric Evaluation of Disability Inventory (Pedi). Esse instrumento de avaliação infantil avalia o desempenho funcional, em termos de capacidades (habilidades funcionais) e desempenho do que a criança realmente faz em resposta ao ambiente (a quantidade de assistência do cuidador necessária para realizar as tarefas diárias) em três domínios: autocuidado, mobilidade e função social. Tal inventário foi traduzido para o português e adaptado culturalmente para contemplar as especificidades socioculturais brasileiras.(25)

O tempo de avaliação será aproximadamente 45 minutos.

**Qualidade de vida**

A qualidade de vida será avaliada através do Questionário de Saúde das Crianças – Relatório dos Pais 50 (Child Health Questionnaire – Parent Form 50 – CHQ-PF50), sendo esta uma ferramenta útil, uma vez que permite avaliar esse aspecto sob a perspectiva dos pais.

O tempo para o responsável pelas crianças de responder o questionário será de aproximadamente 10 minutos.

**Coleta de Saliva**

As amostras de saliva serão coletadas no dia 1 e após 24 sessões entre 10 e 11 horas da manhã, e instruções específicas serão dadas aos participantes, incluindo: evitar escovar os dentes, usar estimulantes salivares e consumir uma refeição principal dentro de 1 h antes da coleta, evitar consumir alimentos ácidos ou com alto teor de açúcar 20 minutos antes da coleta.

O participante, sentado confortavelmente numa cadeira, em ambiente bem ventilado e arejado e com a cabeça ligeiramente inclinada para frente, sem deglutir, permitirá que a saliva se acumule no assoalho da boca sendo levemente expelida em um tubo estéril previamente pesado acoplado a um funil e mergulhado em gelo picado, por um período de 5 minutos.

As amostras salivares serão em seguida transportadas com refrigeração ao laboratório de pesquisa que se encontra na Uninove Vergueiro, terceiro subsolo para serem centrifugadas (5.000 g, 5 min, 4C). As alíquotas de 200 µl serão armazenadas em microtubos no freezer -80º. Após o uso, as amostras serão descartadas.

**Avaliação do BDNF**

A expressão proteica de BDNF será quantificada a partir de amostras de saliva por meio da técnica ELISA. Será utilizado kit comercial DELUXE HUMAN (BioLegend®). A análise será de acordo com as instruções do fabricante. A densidade óptica das amostras será medida no espectrofotômetro a 450 nm.

**Análise estatística**

Os dados serão tabulados e tratados no software GraphPad PRISM versão 7.0. Os valores serão testados quanto a sua normalidade pelo teste Kolmogorov-Smirnov, e serão expressos em média e desvio padrão se assumem a curva de Gauss. Para comparação entre os grupos será realizado ANOVA, considerando um nível de significância de 0,5% (p<0,05).

**References**

1. Copp AJ, Adzick NS, Chitty LS, Fletcher JM, Holmbeck GN, Shaw GM. Spina bifida. Nat Rev Dis Prim. 2015;1:1–45.

2. Kural C, Solmaz I, Tehli O, Temiz C, Kutlay M, Daneyemez MK, et al. Evaluation and Management of Lumbosacral Myelomeningoceles in Children. Eurasian J Med. 2015;47(3):174–8.

3. Marotta M, Fernández-Martín A, Oria M, Fontecha CG, Giné C, Martínez-Ibáñez V, et al. Isolation, characterization, and differentiation of multipotent neural progenitor cells from human cerebrospinal fluid in fetal cystic myelomeningocele. Stem Cell Res [Internet]. 2017;22:33–42. Available from: http://dx.doi.org/10.1016/j.scr.2017.05.003

4. Saadai P, Nout YS, Encinas J, Wang A, Downing TL, Beattie MS, et al. Prenatal repair of myelomeningocele with aligned nanofibrous scaffolds - A pilot study in sheep. J Pediatr Surg [Internet]. 2011;46(12):2279–83. Available from: http://dx.doi.org/10.1016/j.jpedsurg.2011.09.014

5. Zieba J, Miller A, Gordiienko O, Smith GM, Krynska B. Clusters of amniotic fluid cells and their associated early neuroepithelial markers in experimental myelomeningocele: Correlation with astrogliosis. PLoS One. 2017;12(3):1–16.

6. Anderson MA, Burda JE, Ren Y, Ao Y, O’Shea TM, Kawaguchi R, et al. Astrocyte scar formation aids CNS axon regeneration. Nature. 2016;532(7598):195–200.

7. Hoffer, Perry M, Perry ER, Sersie C, Los C. Functional Ambulation Myelomen in Patients i ngocele. 1973;55.

8. Assistência D, Deficiente C, Paulo S, Deficiente C, Paulo S. Ambulation follow-up in patients with myelomeningocele treated at the Associação de Assistência à Criança Deficiente (AACD) in São Paulo, Brazil. 2004;6–9.

9. Aizawa CYP, Morales MP, Lundberg C, Moura MCDS de, Pinto FCG, Voos MC, et al. Conventional physical therapy and physical therapy based on reflex stimulation showed similar results in children with myelomeningocele. Arq Neuropsiquiatr. 2017;75(3):160–6.

10. Ketelaar M, Verschuren O, Gorter JANW, Vos RC, Verheijden J, Jongmans MJ, et al. Efficacy of three therapy approaches in preschool children with cerebral palsy : a randomized controlled trial. 2015;

11. Novak I, Morgan C, Fahey M, Finch-Edmondson M, Galea C, Hines A, et al. State of the Evidence Traffic Lights 2019: Systematic Review of Interventions for Preventing and Treating Children with Cerebral Palsy. Curr Neurol Neurosci Reports 2020 202 [Internet]. 2020;20(2):1–21. Available from: http://link.springer.com/10.1007/s11910-020-1022-z%0Ahttps://link.springer.com/article/10.1007/s11910-020-1022-z?shared-article-renderer

12. Harris SR, Winstein CJ. The Past , Present , and Future of Neurorehabilitation : From NUSTEP Through IV STEP. 2017;

13. Kimberley TJ, Novak I, Boyd L, Fowler E. Stepping Up to Rethink the Future of Rehabilitation : IV STEP Considerations. 2017;

14. Kusiak AN, Selzer ME. Neuroplasticity in the spinal cord [Internet]. 1st ed. Vol. 110, Neurological Rehabilitation. Elsevier B.V.; 2013. 23–42 p. Available from: http://dx.doi.org/10.1016/B978-0-444-52901-5.00003-4

15. Czuba E, Was M, Steliga A, Morys J. BDNF : A Key Factor with Multipotent Impact on Brain Signaling and Synaptic Plasticity. 2018;579–93.

16. Freitas LF De, Hamblin MR. Proposed Mechanisms of Photobiomodulation or Low-Level Light Therapy. 2017;1–37.

17. Hashmi JT, Huang YY, Osmani BZ, Sharma SK, Naeser MA, Hamblin MR. Role of low-level laser therapy in neurorehabilitation. PM R [Internet]. 2010;2(12 SUPPL):S292–305. Available from: http://dx.doi.org/10.1016/j.pmrj.2010.10.013

18. Veronez S, Assis L, Campo P Del, Oliveira F De, Castro G De, Claudia A, et al. Effects of different fluences of low-level laser therapy in an experimental model of spinal cord injury in rats. Lasers Med Sci [Internet]. 2016; Available from: http://dx.doi.org/10.1007/s10103-016-2120-7

19. Byrnes KR, Waynant RW, Ilev IK, Wu X, Barna L, Smith K, et al. Light promotes regeneration and functional recovery and alters the immune response after spinal cord injury. Lasers Surg Med. 2005;36(3):171–85.

20. da Silva FC, Gomes AO, da Costa Palácio PR, Politti F, de Fátima Teixeira da Silva D, Mesquita-Ferrari RA, et al. Photobiomodulation improves motor response in patients with spinal cord injury submitted to electromyographic evaluation: randomized clinical trial. Lasers Med Sci. 2018;33(4):883–90.

21. Cordeiro F, Silva T, Gomes AO, Roberto P, Andreo L, Leticia M, et al. Sensory and motor responses after photobiomodulation associated with physiotherapy in patients with incomplete spinal cord injury : clinical , randomized trial. 2020;

22. Flores MB, Manella KJ, Ardolino EM, Flores MB, Manella KJ, Ardolino EM, et al. Relationship between Movement Quality , Functional Ambulation Status , and Spatiotemporal Gait Parameters in Children with Myelomeningocele Relationship between Movement Quality , Functional Ambulation Status , and Spatiotemporal Gait Parameters in Childr. Phys Occup Ther Pediatr [Internet]. 2020;0(0):1–13. Available from: https://doi.org/10.1080/01942638.2020.1736233

23. Neves A, Visicatto LP, Oliveira AB De, Adriana N, Ferreira C. Effects of Kinesio taping in rectus femoris activity and sit-to-stand movement in children with unilateral cerebral palsy : placebo-controlled , repeated-measure design. Disabil Rehabil [Internet]. 2018;0(0):1–11. Available from: https://doi.org/10.1080/09638288.2018.1458912

24. Hermens HJ. Development of recommendations for SEMG sensors and sensor placement procedures. 2000;10:361–74.

25. Steinhart S, Kornitzer E, Baron AB, Wever C, Shoshan L, Katz-Leurer M. Independence in self-care activities in children with myelomeningocele: exploring factors based on the International Classification of Function model. Disabil Rehabil [Internet]. 2018;40(1):62–8. Available from: http://dx.doi.org/10.1080/09638288.2016.1243158

26. Krakauer JW, Carmichael ST, Corbett D. Getting Neurorehabilitation Right – What Can We Learn From Animal Models? 2015;26(November 2010):923–31.

27. Manuscript A. Axon regeneration and exercise-dependent plasticity after spinal cord injury. 2014;1279(1):154–63.

28. Khan F, Amatya B, Galea MP, Gonzenbach R. Neurorehabilitation : applied neuroplasticity. 2016;

29. Wu X, Dmitriev AE, Cardoso MJ, Viers-costello AG, Borke RC, Streeter J, et al. 810 nm Wavelength Light : An Effective Therapy for Transected or Contused Rat Spinal Cord. 2009;41(October 2008):36–41.

30. Paula AA, Nicolau RA, Lima M de O, Salgado MAC, Cogo JC. “Low-intensity laser therapy effect on the recovery of traumatic spinal cord injury.” Lasers Med Sci. 2014;29(6):1849–59.
