# Supplementary material for: Effect of photobiomodulation combined with physical therapy on functional performance in children with myelomeningocele: A protocol randomized clinical blind study
Source: PLoS One. 2021 Oct 6;16(10):e0253963. doi: 10.1371/journal.pone.0253963 (PMC8494316; doi:10.1371/journal.pone.0253963)
Supplement: S7 File — (DOCX) [file pone.0253963.s007.docx]

**Effects of physiotherapy associated with photobiomodulation on functional performance in children with myelomeningocele- Clinical, randomized and blind study**

**PhD student:** Tamiris da Silva

**Advisor:** Prof. Dr. Sandra Kalil Bussadori

**Institution:** Nove de Julho University

**Graduate Program in Science of**

**Rehabilitation**

Summary

Myelomeningocele is a severe type of spina bifida, resulting from inadequate neural tube closure. This condition drastically affects the structures of the spinal cord as a result, the spinal cord, nerve roots and meninges are exposed during pregnancy, resulting in deficiencies. The combination of these deficiencies results in a general decrease in mobility and functional participation. There is little evidence on physical therapy interventions in this population. However, it is known that the International Classification of Functionality, Disability and Health for Children and Young People is a useful tool to assist therapists in problem analysis and reflection on the focus of the intervention. The current literature demonstrates that resources such as low intensity light, also known as photobiomodulation as a therapeutic medium, can be auxiliary means in the rehabilitation of neurological conditions, because studies show that photobiomoduction promotes sensory and motor recovery in the animal model of spinal cord injury. And a clinical trial showed that after treatment with photobiomodulation combined with photobiomodulation, individuals with spinal cord injury improved motor and sensory function. Therefore, the aim of the study is to evaluate the effects of physiotherapy associated with photobiomodulation on the functional performance of children with low and sacral lumbar myelomeningocele. Materials and methods: will be recruited at the Integrated Health Clinic of The Nove de Julho University. Those who meet the inclusion criteria will be randomized to two groups using a randomization (randomization.com). Group 1 will be submitted to active PBM and physical therapy exercises. Group 2 will be submitted to PBM simulations and physical therapy exercises. Irradiation will be performed with an LED with a wavelength of 850 nm, energy per point of 25 J, 50 seconds per point and power of 200 mW. in the placebo group, the device will not emit light. The objectives of the physiotherapy exercises will be carried out according to the goals and objectives of the child / guardians. the treatment protocol will be twice a week for 24 sessions. evaluations will be performed before treatment, at the last treatment session and 30 days after treatment. Muscle activity will be evaluated using a portable electromyography (BTS Engineering) and, as a measure of functionality, the task of sitting and lifting will be performed. The electrodes will be positioned on the muscles: lateral gastrocnemius, anterior tibial and femoral reto. To assess the functional independence of children, the Pediatric Disability Assessment Inventory will be used. Quality of life will be assessed by the Parents' Health Questionnaire - Form 50. The sensory evaluation will be performed with the Semmes-Weinstein kit (Smiles®). The protein expression of BDNF will be quantified from saliva samples using the ELISA technique. The data will be analyzed with the help of GraphPad PRISM version 7.0.

**Key Words:** Myelomeningocele, Physical Therapy Modalities, Physical Functional Performance, photobiomodulation, Electromyography

**Introduction**

Myelomeningocele (MMC) is a severe type of spina bifida, resulting from inadequate neural tube closure. (1) The multifactorial etiology of CMM is related to environmental and maternal factors. The overall incidence of the disease is one in every 1000 live births. (2) This condition drastically affects the structures of the spinal cord, since the vertebrae at the level of the lesion do not have a spinous process and are therefore incomplete dorsally, this factor causes the spinal cord, nerve roots and meninges to be exposed during pregnancy resulting in deficiencies including paraplegia, skeletal deformities, muscle weakness, loss of sensation, poor coordination, decreased balance, hydrocephalus, Arnold Chiari malformation, and fecal, urinary and sexual dysfunctions. The combination of these deficiencies results in a general decrease in mobility and functional participation. (3)

The pathogenesis of CMM is not fully elucidated, but increasing evidence indicates that spinal cord damage associated with CMM occurs due to the primary cause which is related to abnormal spinal cord development and the secondary cause that occurs due to traumatic and chemical injuries subsequent to spinal cord exposure and are associated with loss of neurological function in fetuses with CMM. (4,5) After an injury to the central nervous system (CNS), there is no recovery of most axons due to regenerative failures that occur after CNS damage, which usually induces permanent disabilities. These permanent sequelae occur because astrocytes form scars in order to restrict inflammation and preserve neural tissue, which is an essential process for healing, on the other hand, these chronic scars are harmful because they continuously prevent the regeneration of the axon. (6)

Thus, the treatment strategies for this condition have the objective of minimizing the extent of the lesion reducing the possible sequelae. The classic treatment for CMM consists of surgical closure of the MMC defect still in the intrauterine phase or shortly after birth. Comparative studies between prenatal and postnatal repair have shown that in both treatments functional impairment below the level of the lesion remains incomplete. (4)

Functional impairment was classified by Hoffer et al. (7) at functional levels according to neurological impairment: thoracic, lumbar high, lumbar low and sacral. The prognosis of ambulation and the objectives to be achieved in rehabilitation depend not only on the neurological level, but also on the presence or not of orthopedic deformities, obesity, cognitive demotion and socio-economic conditions of the family. (8)

In relation to physical therapy interventions, it is known that they focus on objectives of optimizing mobility and maximizing independence and participation, which can be facilitated by muscle strengthening, adaptive positioning and / or better postural control. However, evidence on the efficacy of physiotherapy exercises in children with CMM is limited. (9)

It is known that the International Classification of Functionality, Disability and Health for Children and Young People (CIF-CY) is a useful tool to assist therapists regarding problem analysis and thinking about the focus of the intervention. For a long time, the interventions focused on functional deficiencies and the level of body structure, that is, on the child's abilities. More recently, a change has been observed in the literature towards an approach in which interventions focus on environmental factors, that is, the change of restrictions in a task or in the child's environment, thus emphasizing a more efficient way of completing an activity. (10.11)

The evidence in physical therapy practice for individuals of all ages with neurological disorders, focuses on 5 Ps: prevention, prediction, participation, personalized and plasticity. Therefore, neurological physiotherapists should perform care with a focus on preventing the patient's disabilities, predicting the ideal response of an intervention, through measures of results of the movement system. In addition, it is of paramount importance that the goal of rehabilitation is that people with neurological disabilities are fully included and participate in life activities that are important to them and that their care is personalized. All these measures facilitate the positive plasticity process. (12.13)

Neuroplasticity refers to the tendency of neural circuits to undergo physiological and/or structural changes in response to changes in patterns caused by environmental injuries and/or influences, that is, there is increased angiogenesis and synapses Although neuroplasticity is most commonly associated with the cerebral cortex, all parts of the nervous system, including the spinal cord, demonstrate plasticity, such as the synaptic mechanisms of learning and memory, dendritic pruning, collateral sprouting and axonal regeneration. (14) Another factor that influences and regulates neuroplasticity is the neural growth neutron factor (BDNF), the most important functions of BDNF include, regulation of synaptogenesis, neuroprotection and increased dendritic afforestation, in addition BDNF influences the functional and structural aspects of synaptic transmission. Rehabilitation induces neuroplasticity that can be evidenced by improving functional performance and increasing BDNF. (15)

The current literature demonstrates that resources that are used as a therapeutic means can be used as auxiliaries in the rehabilitation of neurological conditions, such as stroke, neurodegenerative diseases and spinal cord injuries. Photobiomodulation (PBM) occurs from the application of low intensity light (red and infrared light), such as low-intensity laser and LED in biological tissues. The therapeutic efficacy of phototherapy is based on the absorption of photons by photoreceptors or chromophores. (16.17)

Studies in the experimental model of spinal cord injury, the authors demonstrated that both red and infrared wavelength have the potential to be effective and noninvasive means of therapy, promoting axonal budding, increased concentration of glial cells and nerve connections, and functional and sensory improvement. (18,19) The findings of a clinical trial involving individuals with a diagnosis of spinal cord injury demonstrate that PBM exerted positive effects on motor function, especially during isotonic contraction of stimulated muscles evaluated by electromyography (EMG). (20) In addition, Silva et al. (21) demonstrated that after 12 photobiomodulation sessions associated with physiotherapy in patients with spinal cord injury, there was recovery in sensory perception and muscle strength. Therefore, FBM may be a promising treatment associated with physical therapy exercises with CMM.

**Justification**

The present study proposes to conduct a controlled clinical trial to evaluate the effectiveness of physiotherapy associated with FBM as a complementary treatment in sensory and motor improvement in individuals with CMM. THE FBM is a noninvasive treatment, and a rapid procedure and can be a promising approach in the treatment, but the literature has shown little studies on the subject. MMC, as well as in the edular mlesion, is a severe trauma of the nervous system and there are still no effective restorative therapies, however, FBM has biomodulatory effects on the tissue of the central and peripheral nervous system.

If there are positive results, it is possible to propose a new form of treatment for the sensormotor response in these individuals.

**Objective**

Evaluate the functional performance of children with myelomeningocele after physiotherapy exercises associated with photobiomodulation.

**Secondary Objectives**

-Assess surface sensitivities with Semmes-Weinstein monofilaments

- Assess functional independence through the PEDI scale

- Assess quality of life through Children's Health Questionnaire - Parents Report 50

- Evaluate the protein expression of BDNF levels in saliva samples by ELISA

**Materials and Methods**

This study will be a randomized, controlled clinical trial that will be developed according to the flowchart presented in Figure 1.

This study will follow the regulatory standards of research in human beings with submission and approval of the Ethics Committee (CNS no. 466/12 and Res. CNS 510/2016) in research from The Nove de Julho University.

After the approval of the ethics committee, an invitation to participate in the research will be held after the survey of patients who undergo physiotherapy or are on the waiting list of the UNINOVE outpatient clinic. Participants will be invited to carry out the screening, in which the initial evaluation form will be applied (Annex1). Participants or guardians will sign the Free and Informed Consent Form (TCLE) (Annex 2)and children aged5-14 years will sign the consent form (Annex 3 5). In addition, children who do not know how to read, researchers, will read, explain through the images every procedure that will be performed, and in the end if the children agree, researchers will put ink on the children's finger so that they sign whether or not to participate in the research.

R crutising

Included

Deleted

Group 1

Physiotherapy exercises

+

Active photobiomodulation

Group 2

Physiotherapy exercises

+

Photobiomodulation Placebo

Evaluations

T0= pre-intervention

T1= after 12 sessions of PBM physiotherapy + post-intervention

T2= 30 days after completion of the intervention (follow-up)

Figura 1. Study design.

**Participantes**

Eligibility to participate in the study will depend on the following criteria:

**Inclusion criteria: age** from 5 to 14 years; diagnosis of myelomeningocele at the lower lumbar and sacral level; Get Director or sit-to-stand Movement.

**Exclusion criteria: cognitive** impairment that compromises the ability to communicate and answer the questions that will be posed; latex allergy; secondary manifestations to CMM, such as congenital lye foot; neuromuscular scoliosis; subluxation or dislocation of the hip and knee; other central nervous system disease.

**Randomization**

Participants will be randomized into two groups using a randomization (randomization.com) site. Group 1 will undergo active photobiomodulation and physical therapy exercises. Group 2 will be submitted to photobiomodulation simulations and physical therapy exercises.

**Blinding**

- Participants will not know if which group they are participating in, i.e. treatment group or placebo with photobiomodulation.
- The evaluator and who will perform the exercises will not know which group the participant is participating in relation to photobiomodulation.
- A therapist who will not participate in the evaluations and sessions of physiotherapy will perform the application of photobiomodulation.
- The microtubes with the saliva samples will be marked with numbers corresponding to each participant, so the evaluator will not know which group the sample corresponds to. A therapist will be responsible for the evaluation and electromyography scales.

**Interventions**

**Photobiomodulation protocol**

For irradiation the individuals will be comfortably positioned in lateral decubitus on the stretcher. It will be radiated only 4 points, on top of the level of the injury. The level of the injury will be located through the imaging examination, which will be requested for the person responsible to bring on the day of the evaluation. After determining the level of the lesion, the transverse processes of the vertebrae will be performed.

The same LED device will be used for both groups, however, in the placebo group the laser device will emit sound that will outline the start and end of therapy, but will not emit light. Twelve sessions will be held twice a week using the parameters described in Table 1.

Twenty-four sessions will be held twice a week.

| **Parameters** | **Infrared LED** |
| --- | --- |
| Wavelength [nm] | 850 |
| Operation mode | Continuous |
| Diameter [cm] | 0,4 |
| Area [cm^2^] | 0,126 |
| Irradiance [W/cm^2^] | 796 |
| Duration of exposure [s] | 50 (per point) |
| Radiant exposure [J/cm^2^] | 200 |
| Radiant energy [J] | 25 (per point) |
| Number of irradiated points | 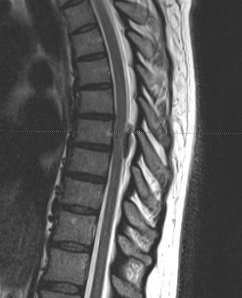4 |
| Application technique | Contact |
| Number and frequency of sessions  treatment | 2 times a week  24 sessions |

**Physical therapy**

Physiotherapy exercises will be individualized and customized for each child. It will take into account complaints and/or engine delays arising from the injury. Circuit exercises involving muscle strengthening exercises, sensory stimulation and balance will be performed. All exercises will be associated with recreational activities such as playing ball, fishing. Participants can also get involved in the story of princesses and superheroes that allow them to perform tasks related to objectives.

Physiotherapy will be twice a week for 45-60 minutes after the application of the LED for 24 sessions

**Criteria for discontinuing intervention**

Faltar for 2 consecutive times or have 3 non-consecutive fouls should discontinue the intervention.

**Aderence**

All interventions are performed individually. To ensure compliance with the predefined protocol, physiotherapists will be instructed by the principal investigator on how to provide the interventions, as well as receive guidance from the exercises. Therapists will record how was the training in terms of type and execution of exercises, intensity level and repetitions. The physiotherapist responsible for the intervention in the participating physiotherapy clinic records symptoms of possible adverse events related to treatment. Any adverse event is then reported to the principal investigator, who then reports to the regional ethics committee.

**Evaluations**

The evaluations will be performed before therapy (pre-intervention-T0), after 12 sessions of physiotherapy PBM + (post-intervention) (T1) and 30 days after the completion of the intervention (follow-up) (T2).

**Surface electromyography**

Muscle activity will be evaluated using a portable electromyographic (BTS Engineering) synchronized to the BST EMG analizer system. As a measure of functionality will be performed the task sit-to-stand. (22.23)

The electrodes will be positioned in the muscles: lateral gastrocnemius (GL), tibial anterior (AT) and femoral reto (RF).

Skin preparation and electrode placement will be in accordance with noninvasive assessment guidelines (SENIAN). (24) The electrodes will be positioned with the child in ventral decubitus (GL region) and dorsal decubitus (TA and RF region)

For the evaluation of the sit-to-stand the participant will be positioned in a chair with 90º of hip flexion, knee, ankle and feet supported.

An evaluation will be performed with the child sitting at rest. After you will be asked to stand up and remain in an orthostatism position for 10 seconds. After you will be asked to sit down. The sit-to-stand task will be repeated 3 times with an interval of 5 minutes.

Children will perform the movement at the speed they usually adopt in their daily routine.

The collection time will be approximately 30 minutes,always respecting a rest interval between each collection, to avoid the effects of fatigue.

**Sensory assessment**

The sensory evaluation will be performed with the Semmes-Weinstein kit. The kit contains a set of six nylon monofilaments (esthesiometry) of the same length, which exert force on the specific area tested. Each monofilament is represented by a color and diameter: Green (0.05 g), blue (0.2 g), violet (2 g), red (4 g), orange (10 g) and magenta red (300 g) 16. The test will be performed on the dermatomes (Scores ranged from seven (green monofilament) to 1 (magenta red monofilament). ^18th^

The following dermatomes will be evaluated: L1, L2, L4, L5,S1, S2.

The evaluation time will beapproximately 15 minutes.

**Participation and Environmental Measure**

The change in participation will be evaluated using the Measure of Participation and Environment for Children and Young People (PEM-CY). The PEM-CY is a parent reporting tool that examines participation and environmental factors that affect children's participation in three environments: home, school and community. Parents will be asked to evaluate their children's involvement in 25 activities in the three environments. For each activity, the parent will ask how often their child participated in one or more such activities and the question will be evaluated on an 8-point scale, from never to daily. In addition, they will be asked to evaluate their children's involvement in each of the activities using a 5-point scale, from minimally involved to very involved, and whether they want their children's participation to change in this type of activity. (26)

**functional abilities**

The Pediatric Evaluation of Disability Inventory (Pedi) will be used to assess the functional independence of children. This child assessment instrument evaluates functional performance in terms of skills (functional skills) and performance of what the child actually does in response to the environment (the amount of caregiver assistance needed to perform daily tasks) in three domains: self-care, mobility and social function. This inventory was translated into the Portuguese and culturally adapted to contemplate the Brazilian sociocultural specificities. (25)

The evaluation time will be approximately 45 minutes.

**Quality of life**

Quality of life will be assessed through the Children's Health Questionnaire 50 (Child Health Questionnaire - Parent Form 50 - CHQ-PF50), which is a useful tool, since it allows evaluating this aspect from the perspective of parents.

The time for the person responsible for the children to answer the questionnaire will be approximately 10 minutes.

**Saliva Collection**

Saliva samples will be collected on day 1 and after 24if sonbetween 10 and 11 a.m., and specific instructions will be given to participants, including: avoid brushing teeth, using salivary stimulants and consuming a main meal within 1 h before collection, avoiding consuming acidic foods or with high sugar content 20 minutes before collection.

The participant, sitting comfortably in a chair, in a well ventilated and airy environment and with his head slightly tilted forward, without swallowing, will allow saliva to accumulate on the floor of the mouth being lightly expelled in a previously heavy sterile tube coupled to a funnel and dipped in crushed ice for a period of 5 minutes.

The salivary samples will then be transported with refrigeration to the research laboratory located in Uninove Vergueiro, third subsoil to be centrifuged (5,000 g, 5 min, 4C). The aliquots of 200 μl will be stored in microtubes in the freezer -80º. After use, the samples will be discarded.

**BDNF Evaluation**

The protein expression of BDNF will be quantified from saliva samples using the ELISA technique. Deluxe HUMAN commercial kit (BioLegend®) will be used. The analysis will be according to the manufacturer's instructions. The optical density of the samples will be measured in the spectrophotometer at 450 nm.

**Statistical analysis**

The data will be tabulated and processed in the GraphPad PRISM software version 7.0. The values will be tested for their normality by the serão Kolmogorov-Smirnov test, and will be expressed as average and standard deviation if they assume the Gauss curve. For comparison between the groups, theANOVA will be performed,considering a significance level of 0.5% (p<0.05).

**References**

1. AJ Copp, Adzick NS, Chitty LS, Fletcher JM, Holmbeck GN, GM Shaw. Spina bifida. Nat Rev Dis Prim. 2015;1:1–45.

2. Kural C, Solmaz I, Tehli O, Temiz C, Kutlay M, Daneyemez MK, et al. Evaluation and Management of Lumbosacral Myelomeningoceles in Children. Eurasian J Med. 2015;47(3):174–8.

3. Marotta M, Fernández-Martín A, Oria M, Fontecha CG, Giné C, Martínez-Ibáñez V, et al. Isolation, characterization, and differentiation of multipotent neural progenitor cells from human cerebrospinal fluid in fetal cystic myelomeningocele. Stem Cell Res [Internet]. 2017;22:33–42. Available from: http://dx.doi.org/10.1016/j.scr.2017.05.003

4. Saadai P, Nout YS, Encinas J, Wang A, Downing TL, Beattie MS, et al. Prenatal repair of myelomeningocele with aligned nanofibrous scaffolds - A pilot study in sheep. J Pediatr Surg [Internet]. 2011;46(12):2279–83. Available from: http://dx.doi.org/10.1016/j.jpedsurg.2011.09.014

5. Zieba J, Miller A, Gordiienko O, Smith GM, Krynska B. Clusters of amniotic fluid cells and their associated early neuroepithelial markers in experimental myelomeningocele: Correlation with astrogliosis. PLoS One. 2017;12(3):1–16.

6. Anderson MA, Burda JE, Ren Y, Ao Y, O'Shea TM, Kawaguchi R, et al. Astrocyte scar formation aids CNS axon regeneration. Nature, nature. 2016;532(7598):195–200.

7. Hoffer, Perry M, Perry ER, Sersie C, Los C. Functional Ambulation Myelomen in Patients i ngocele. 1973;55.

8. Assistance D, Disabled C, Paulo S, Disabled C, Paulo S. Ambulation follow-up in patients with myelomeningocele treated at the Association of Assistance to Disabled Children (AACD) in São Paulo, Brazil. 2004;6–9.

9. Aizawa CYP, Morales MP, Lundberg C, Moura MCDS de, Pinto FCG, Voos MC, et al. Conventional physical therapy and physical therapy based on reflex stimulation showed similar results in children with myelomeningocele. Arq Neuropsiquiatr. 2017;75(3):160–6.

10. Ketelaar M, Verschuren O, Gorter JANW, Vos RC, Verheijden J, Jongmans MJ, et al. Efficacy of three therapy approaches in preschool children with cerebral palsy : a randomized controlled trial. 2015;

11. Novak I, Morgan C, Fahey M, Finch-Edmondson M, Galea C, Hines A, et al. State of the Evidence Traffic Lights 2019: Systematic Review of Interventions for Preventing and Treating Children with Cerebral Palsy. Curr Neurol Neurosci Reports 2020 202 [Internet]. 2020;20(2):1–21. Available from: http://link.springer.com/10.1007/s11910-020-1022-z%0Ahttps://link.springer.com/article/10.1007/s11910-020-1022-z?shared-article-renderer

12. Harris SR, CJ Winstein. The Past , Present , and Future of Neurorehabilitation : From NUSTEP Through IV STEP. 2017;

13. Kimberley TJ, Novak I, Boyd L, Fowler E. Stepping Up to Rethink the Future of Rehabilitation : IV STEP Considerations. 2017;

14. Kusiak AN, Selzer ME. Neuroplasticity in the spinal cord [Internet]. 1st ed. Vol. 110, Neurological Rehabilitation. B.V. Elsevier; 2013. 23–42 p. Available from: http://dx.doi.org/10.1016/B978-0-444-52901-5.00003-4

15. Czuba E, Was M, Steliga A, Morys J. BDNF : A Key Factor with Multipotent Impact on Brain Signaling and Synaptic Plasticity. 2018;579–93.

16. Freitas LF De, Hamblin MR. Proposed Mechanisms of Photobiomodulation or Low-Level Light Therapy. 2017;1–37.

17. Hashmi JT, Huang YY, Osmani BZ, Sharma SK, Naeser MA, Hamblin MR. Role of low-level laser therapy in neurorehabilitation. PM R [Internet]. 2010;2(12 SUPPL):S292–305. Available from: http://dx.doi.org/10.1016/j.pmrj.2010.10.013

18. Veronez S, Assis L, Campo P Del, Oliveira F De, Castro G De, Claudia A, et al. Effects of different fluences of low-level laser therapy in an experimental model of spinal cord injury in rats. Lasers Med Sci [Internet]. 2016; Available from: http://dx.doi.org/10.1007/s10103-016-2120-7

19. Byrnes KR, Waynant RW, Ik Ilev, Wu X, Barna L, Smith K, et al. Light promotes regeneration and functional recovery and alters the immune response after spinal cord injury. Lasers Surg Med. 2005;36(3):171–85.

20. da Silva FC, Gomes AO, da Costa Palácio PR, Politti F, de Fátima Teixeira da Silva D, Mesquita-Ferrari RA, et al. Photobiomodulation improves motor response in patients with spinal cord injury submitted to electromyographic evaluation: randomized clinical trial. Lasers Med Sci. 2018;33(4):883–90.

21. Cordeiro F, Silva T, Gomes AO, Roberto P, Andreo L, Leticia M, et al. Sensory and motor responses after photobiomodulation associated with physiotherapy in patients with incomplete spinal cord injury : clinical , randomized trial. 2020;

22. Flowers MB, Manella KJ, Ardolino EM, Flores MB, Manella KJ, Ardolino EM, et al. Relationship between Movement Quality , Functional Ambulation Status , and Spatiotemporal Gait Parameters in Children with Myelomeningocele Relationship between Movement Quality , Functional Ambulation Status , and Spatiotemporal Gait Parameters in Childr. Phys Occup Ther Pediatr [Internet]. 2020;0(0):1–13. Available from: https://doi.org/10.1080/01942638.2020.1736233

23. Neves A, Visicatto LP, Oliveira AB De, Adriana N, Ferreira C. Effects of Kinesio taping in rectus femoris activity and sit-to-stand movement in children with unilateral cerebral palsy : placebo-controlled , repeated-measure design. Disabil Rehabil [Internet]. 2018;0(0):1–11. Available from: https://doi.org/10.1080/09638288.2018.1458912

24. HJ Hermens. Development of recommendations for SEMG sensors and sensor placement procedures. 2000;10:361–74.

25. Steinhart S, Kornitzer E, Baron AB, Wever C, Shoshan L, Katz-Leurer M. Independence in self-care activities in children with myelomeningocele: exploring factors based on the International Classification of model Function. Disabil Rehabil [Internet]. 2018;40(1):62–8. Available from: http://dx.doi.org/10.1080/09638288.2016.1243158

26. Krakauer JW, Carmichael ST, Corbett D. Getting Neurorehabilitation Right – What Can We Learn From Animal Models? 2015;26(November 2010):923–31.

27. Manuscript A. Axon regeneration and exercise-dependent plasticity after spinal cord injury. 2014;1279(1):154–63.

28. Khan F, Amatya B, Galea MP, Gonzenbach R. Neurorehabilitation : applied neuroplasticity. 2016;

29. Wu X, Dmitriev AE, Cardoso MJ, Viers-costello AG, Borke RC, Streeter J, et al. 810 nm Wavelength Light : An Effective Therapy for Transected or Contused Rat Spinal Cord. 2009;41(October 2008):36–41.

30. Paula AA, Nicolau RA, Lima M de O, Salgado MAC, Cogo JC. "Low-intensity laser therapy effect on the recovery of traumatic spinal cord injury." Lasers Med Sci. 2014;29(6):1849–59.
