# Supplementary material for: Effect of photobiomodulation combined with physical therapy on functional performance in children with myelomeningocele: A protocol randomized clinical blind study
Source: PLoS One. 2021 Oct 6;16(10):e0253963. doi: 10.1371/journal.pone.0253963 (PMC8494316; doi:10.1371/journal.pone.0253963)
Supplement: S8 File — (PDF) [file pone.0253963.s008.pdf]

PARECER CONSUBSTANCIADO DO CEP

DADOS DO PROJETO DE PESQUISA

Título da Pesquisa: Efeitos da fisioterapia associada com a fotobiomodulação no desempenho funcional em crianças com mielomeningocele- Estudo clínico, randomizado e cego

Pesquisador: TAMIRIS DA SILVA
Área Temática:
Versão: 3
CAAE: 33626720.0.0000.5511
Instituição Proponente: ASSOCIACAO EDUCACIONAL NOVE DE JULHO
Patrocinador Principal: Financiamento Próprio

DADOS DO PARECER

Número do Parecer: 4.308.134

Apresentação do Projeto:
Informações extraídas do projeto:
Título da Pesquisa: Efeitos da fisioterapia associada com a fotobiomodulação no desempenho funcional em crianças com mielomeningocele- Estudo clínico, randomizado e cego

Versão:3
CAAE:33626720.0.0000.5511

Apresentação do Projeto: resumo
A mielomeningocele é um tipo grave de espinha bífida, resultante do fechamento inadequado do tubo neural. Essa condição afeta drasticamente as estruturas da medula espinhal, como resultado, a medula espinhal, as raízes nervosas e as meninges são expostas durante a gravidez, resultando em deficiências. A combinação dessas deficiências resulta em uma diminuição geral da mobilidade e da participação funcional. Há poucas evidências sobre intervenções fisioterapêuticas nessa população. No entanto, sabe-se que a Classificação Internacional de Funcionalidade, Incapacidade e Saúde para Crianças e Jovens é uma ferramenta útil para auxiliar os terapeutas na análise de problemas e na reflexão sobre o foco da intervenção. A literatura atual demonstra que recursos como a luz de baixa intensidade, também conhecida como fotobiomodulação como meio

terapêutico, podem ser meios auxiliares na reabilitação de condições neurológicas, pois estudos mostram que a fotobiomodução promove recuperação sensorial e motora no modelo animal de lesão medular . E um ensaio clínico mostrou que, após o tratamento com fotobiomodulação combinado com a fotobiomodulação, indivíduos com lesão medular melhoraram a função motora esensorial. Portanto, o objetivo do estudo é avaliar os efeitos da fisioterapia associada à fotobiomodulação no desempenho funcional de crianças com mielomeningocele lombar baixa e sacral.

Introdução:
A mielomeningocele (MMC) é um tipo grave de espinha bífida, resultante do fechamento inadequado do tubo neural.(1) A etiologia multifatorial da MMC está relacionada a fatores ambientais e maternos. A incidência global da doença é de um em cada 1000 nascidos vivos.(2) Essa condição afeta drasticamente as estruturas da medula espinhal, visto que as vértebras ao nível da lesão não têm processo espinhoso sendo portanto incompletas dorsalmente, este fator faz com que a medula espinhal, raízes nervosas e meninges sejam expostas durante a gestação resultando em deficiências incluindo paraplegia, deformidades esqueléticas, fraqueza muscular, perda de sensação, coordenação deficiente, equilíbrio diminuído, hidrocefalia, malformação de Arnold Chiari, e disfunções fecal, urinária e sexual. A combinação dessas deficiências resulta em uma diminuição geral da mobilidade e participação funcional. (3) A patogênese da MMC não está totalmente elucidada, porém evidências crescentes indicam que os danos na medula espinhal associados a MMC ocorram devido a causa primária a qual está relacionada ao desenvolvimento anormal da medula espinhal e a causa secundária que ocorre devido as lesões traumáticas e químicas subsequentes da exposição da medula e estão associados à perda da função neurológica em fetos com MMC. (4,5) Após uma lesão no sistema nervoso central (SNC), não há recuperação da maioria dos axônios, em virtude de falhas regenerativas que ocorrem após danos no SNC, o que geralmente induz a incapacidades permanentes. Estas sequelas permanentes ocorrem porque os astrócitos formam cicatrizes com objetivo de restringir a inflamação e preservar o tecido neural, sendo este um processo essencial para a cicatrização, por outro lado, essas cicatrizes crônicas são prejudiciais pois impedem continuamente a regeneração do axônio.(6) Sendo assim, as estratégias de tratamento para essa condição possuem o objetivo de minimizar a extensão da lesão reduzindo as possíveis sequelas. O tratamento clássico para a MMC consiste no fechamento cirúrgico do defeito da MMC ainda na fase intrauterina ou logo após o nascimento. Estudos comparativos entre o reparo pré-natal versus o pós-natal demonstraram que em ambos os tratamentos o

comprometimento funcional abaixo do nível da lesão permanece incompleta.(4) O comprometimento funcional foi classificado por Hoffer et al.(7) em níveis funcionais de acordo com o comprometimento neurológico: torácico, lombar alto, lombar baixo e sacral. O prognóstico de deambulação e os objetivos a serem alcançados na reabilitação dependem não somente do nível neurológico, mas também da presença ou não de deformidades ortopédicas, obesidade, rebaixamento do cognitivo e condições sócio-econômicas da família.(8) Em relação as intervenções fisioterapêuticas, tem se o conhecimento de que se concentram em objetivos de otimizar a mobilidade e maximizar a independência e participação, o que pode ser facilitado pelo fortalecimento muscular, posicionamento adaptativo e / ou melhor controle postural. No entanto, as evidências sobre a eficácia dos exercícios de fisioterapia em crianças com MMC são limitadas.(9) Sabe-se que a Classificação Internacional de Funcionalidade, Incapacidade e Saúde para Crianças e Jovens (CIF-CY) é uma ferramenta útil para auxiliar os terapeutas quanto a análise de problemas e no pensamento sobre o foco da intervenção. Durante muito tempo, as intervenções concentraram-se nas deficiências funcionais e no nível da estrutura do corpo, ou seja, nas habilidades da criança. Mais recentemente, uma mudança foi observada na literatura em direção a uma abordagem na qual as intervenções se concentram nos fatores ambientais, ou seja, na alteração de restrições em uma tarefa ou no ambiente da criança, enfatizando desta forma mais eficiente de concluir uma atividade. (10,11)As evidências em prática de fisioterapia para indivíduos de todas as idades com distúrbios neurológicos, foca em 5 Ps: prevenção, predição, participação, personalizado e plasticidade. Sendo assim, os fisioterapeutas neurológicos devem realizar o atendimento com foco em prevenir asincapacidades do paciente, prever a resposta ideal de uma intervenção, através de medidas de resultados do sistema do movimento. Além disso, é de suma importância que o objetivo da reabilitação seja que as pessoas com deficiências neurológicas sejam totalmente incluídas e participem de atividades da vida que são importantes para elas e que seu atendimento seja personalizado. Todas essas medidas facilitam o processo de

plasticidade positiva. (12,13) A neuroplasticidade refere-se à tendência dos circuitos neurais de sofrerem alterações fisiológicas e/ou estruturais em resposta a mudanças nos padrões provocadas por lesões e/ou influências ambientais, ou seja, ocorre aumento da angiogênese e sinapses Embora a neuroplasticidade seja mais comumente associada ao córtex cerebral, todas as partes do sistema nervoso, incluindo a medula espinhal, demonstram plasticidade, a exemplo dos mecanismos sinápticos de aprendizado e memória, poda dendrítica, brotação colateral e regeneração axonal.(14) Outro fator que influencia e regula a neuroplasticidade é o fator neutrófico de crescimento neural (BDNF), as funções mais importantes do BDNF incluem, regulação

da sinaptogênese, neuroproteção e aumento da arborização dendrítica, além disso o BDNF influencia os aspectos funcionais e estruturais da transmissão sináptica. A reabilitação induz a neuroplasticidade que pode ser evidenciada através da melhora do
desempenho funcional e aumento de BDNF. (15)A literatura atual demonstra que recursos que se utilizem da luz como meio terapêutico podem ser utilizados como auxiliares na reabilitação de condições neurológicas, a exemplo acidente vascular cerebral, doenças neurodegenerativas e lesões da medula espinhal. A Fotobiomodulação (PBM) ocorre a partir da aplicação de luz de baixa intensidade (luz vermelha e infravermelha), a exemplo do laser de baixa intensidade e diodo emissor de luz (LED) em tecidos biológicos. A eficácia terapêutica da Fototerapia é baseada na absorção de
fótons por fotorreceptores ou cromóforos. (16,17) Estudos no modelo experimental de lesão medular, os autores demonstraram que tanto o comprimento de onda vermelho e o infravermelho têm o potencial de serem meios eficazes e não invasivos, de terapia, promovendo o brotamento axonal, aumento na concentração de células gliais e de conexões nervosas, além de melhora funcional e sensitiva. (18,19) Os achados de um
ensaio clínico envolvendo indivíduos com uma o diagnóstico de lesão medular demonstra que a PBM exerceu efeitos positivos na função motora, principalmente durante a contração isotônica dos músculos estimulados avaliados por eletromiografia (EMG).(20) Além disso, Silva et al.(21) demonstraram que após 12 sessões de fotobiomodulação associado a fisioterapia em pacientes com lesão medular, houve recuperação na
percepção sensorial e força muscular. Portanto, a FBM pode ser um tratamento promissor associado aos exercícios de fisioterapia com MMC. Sendo assim, o objetivo do estudo é avaliar os efeitos da fisioterapia associados a fotobiomodulação na resposta sensorial e motora de crianças com mielomeningocele lombar baixo e sacral.

Materiais e métodos:
serão recrutados na Clínica Integrada de Saúde da Universidade Nove de Julho. Aqueles que atenderem aos critérios de inclusão serão randomizados para dois grupos usando um site de randomização (randomization.com). O grupo 1 será submetido a PBM ativo e exercícios fisioterapêuticos. O grupo 2 será submetido a simulações de PBM e exercícios fisioterapêuticos. A irradiação será realizada com um LED com comprimento de onda de 850 nm, energia por ponto de 25 J, 50 segundos por ponto e potência de 200

mW. no grupo placebo, o dispositivo não emitirá luz. Os objetivos dos exercícios de fisioterapia serão realizados de acordo com as metas e objetivos da criança / responsáveis. o protocolo de tratamento será duas vezes na semana por 24 sessões. as avaliações serão executadas antes do tratamento, na última sessão de tratamento e 30

dias após o tratamento.A atividade muscular será avaliada usando uma eletromiografia portátil (BTS Engineering) e, como medida de funcionalidade, a tarefa de sentar e levantar será realizada. Os eletrodos serão posicionados sobre os músculos: gastrocnêmio lateral, tibial anterior e reto femoral. Para avaliar a independência funcional das crianças, será utilizado o Inventário de Avaliação Pediátrica da Incapacidade. A qualidade de vida será avaliada pelo Questionário de Saúde da Criança - Formulário 50 dos pais. A avaliação sensorial será realizada com o kit Semmes- Weinstein (Smiles®). A expressão proteica do BDNF será quantificada a partir de amostras de saliva usando a técnica ELISA. Os dados serão analisados com o auxílio do GraphPad PRISM versão 7.0.

Hipótese:

H0: Não há diferenças no desempenho funcional após tratamento com fisioterapia associada a fotobiomodulaçãoH1: Há diferenças no desempenho funcional após tratamento com fisioterapia associada a fotobiomodulação.

Tamanho da amostra: 30

Objetivo da Pesquisa:

Objetivo Primário:

Avaliar o desempenho funcional de crianças com mielomeningocele após exercícios de fisioterapia associada a fotobiomodulação.

Objetivo Secundário:

-Avaliar a sensibilidades superficial com os monofilamentos Semmes-Weinstein;

- Avaliar a independência funcional através da escala PEDI;
- Avaliar a qualidade de vida através Questionário de Saúde das Crianças;

– Relatório dos Pais 50- Avaliar a expressão proteica dos níveis de BDNF nas amostras de saliva por ELISA.

Critério de Inclusão:
-idade de 5 a 14 anos;
- diagnóstico de mielomeningocele no nível lombar e sacral inferior;
- Consiga Realizador ou Movimento de Sentar e Levantar
com Apoio.

Critério de Exclusão:
- comprometimento cognitivo que compromete a capacidade de se comunicar e responder às perguntas que serão colocadas;
- alergia ao látex;
- manifestações secundárias ao MMC, como pé torto congênito; escoliose neuromuscular; subluxação ou luxação do quadril e joelho;
- outra doença do sistema nervoso central.

Avaliação dos Riscos e Benefícios:
Desconforto ou Riscos Esperados: Pode ser desconfortável para a criança ficar deitada para aplicação da luz de baixa intensidade por 5 minutos. Lembrando que mesmo que mínimos, sempre existem riscos. Os dias de avaliação da eletromiografia podem ser desconfortáveis para a criança, pois o tempo de avaliação é de 30 minutos, e a criança poderá se sentir impaciente. Também pode ser desconfortável a coleta de saliva, pois a criança precisa cuspir no tubinho.

Benefícios da Pesquisa: A criança irá realizar fisioterapia, os exercícios vão ser de acordo com o objetivo funcional e q queixa principal da criança/responsável.

Medidas protetivas aos riscos: A criança será posicionada de forma confortável para aplicação da luz de baixa intensidade. Na coleta da saliva a criança irá cuspir em um tubinho novo e estéril, além disso os profissionais vão fazer uso de luvas e aventais. Todos os brinquedos utilizados para o desenvolvimento dos exercícios de fisioterapia serão devidamente limpos. O aparelho da luz também será coberto com plástico para evitar o contato direto com a pele.

Comentários e Considerações sobre a Pesquisa:

Trata-se de um ensaio clínico em que 30 crianças (5 a 14 anos de idade) com mielomeningocele que serão randomizadas em dois grupos: (1) terapia de fotobiomodulação associada a exercícios fisioterapêuticos e (2) placebo da fotobiomodulação associado a exercícios fisioterapêuticos. Os exercícios Serão realizadas duas sessões por semana, totalizando 24 sessões. As avaliações serão: a atividade muscular através da eletromiografia de superfície dos músculos gastrocnêmio lateral, tibial anterior e reto femoral; a funcionalidade através da tarefa de sentar e levantar; a independência funcional através do Inventário de Avaliação Pediátrica da Incapacidade; a qualidade de vida através do Questionário de Saúde da Criança - Formulário 50 dos pais; a parte sensorial através do kit Semmes-Weinstein (Smiles®) e a expressão proteica de BDNF quantificada a partir de amostras de saliva usando a técnica ELISA.

As avaliações serão realizadas antes, após as 24 sessões e um mês após o término das intervenções.

Considerações sobre os Termos de apresentação obrigatória:

O presente projeto não apresenta impedimentos éticos.

As solicitações de ajustes foram atendidas, a saber:

- A folha de rosto e as informações básicas do projeto estavam adequadas nas versões anteriores;
- Cronograma: não especifica o ano de realização, mas o projeto indica que a pesquisa se iniciará somente após aprovação do CEP.

As seguintes pendências foram também levantadas na primeira avaliação, com solicitação de:

1- Substituir os termos científicos para uma linguagem que o leigo possa compreender: Parcialmente atendido - permanecem alguns termos de difícil entendimento para o leigo, como "resposta sensorial e motora". ATENDIDA.

2 - No item 4 (Procedimentos da Fase Experimental), acrescentar que se a criança faltar por 2 vezes consecutivas ou tiver 3 faltas não consecutivas deverá descontinuar a intervenção - Não foi acrescentado. ATENDIDA.

Recomendações:

Recomenda-se corrigir no TCLE a frase:

"A participação da criança poderá contribuir para a ampliação do conhecimento sobre a mielomeningocele, e o efeito doa luz em baixa intensidade associado a fisioterapia na força muscular e sensibilidade.", por "A participação da criança poderá contribuir para a ampliação do conhecimento sobre a mielomeningocele, e o efeito da luz em baixa intensidade associado a fisioterapia na força muscular e sensibilidade."

Conclusões ou Pendências e Lista de Inadequações:

Todas as solicitações foram adequadamente atendidas.

Considerações Finais a critério do CEP:

O pesquisador deverá se apresentar na instituição de realização da pesquisa (que autorizou a realização do estudo) para início da coleta dos dados.

O participante da pesquisa (ou seu representante) e o pesquisador responsável deverão rubricar todas as folhas do Termo de Consentimento Livre e Esclarecido - TCLE apondo sua assinatura na última página do referido Termo, conforme Carta Circular no 003/2011 da CONEP/CNS.

Salientamos que o pesquisador deve desenvolver a pesquisa conforme delineada no protocolo aprovado.

Eventuais modificações ou emendas ao protocolo devem ser apresentadas ao CEP de forma clara e sucinta, identificando a parte do protocolo a ser modificada e suas justificativas. Lembramos que esta modificação necessitará de aprovação ética do CEP antes de ser implementada. De forma objetiva com justificativa para nova apreciação, os documentos alterados devem ser evidenciados para facilitar a nova análise.

Ao pesquisador cabe manter em arquivo, sob sua guarda, por 5 anos, os dados da pesquisa, contendo fichas individuais e todos os demais documentos recomendados pelo CEP (Res. CNS 466/12 item X1. 2. f).

De acordo com a Res. CNS 466/12, X.3.b), o pesquisador deve apresentar a este CEP/SMS os relatórios semestrais. O relatório final deverá ser enviado através da Plataforma Brasil, ícone Notificação. Uma cópia digital do projeto finalizado deverá ser enviada à instância que autorizou a

realização do estudo, via correio, e-mail ou entregue pessoalmente, logo que o mesmo estiver concluído.

Este parecer foi elaborado baseado nos documentos abaixo relacionados:

Tipo Documento	Arquivo	Postagem	Autor	Situação
Informações Básicas do Projeto	PB_INFORMAÇÕES_BÁSICAS_DO_PROJETO_1573599.pdf	02/09/2020 16:18:19		Aceito
TCLE / Termos de Assentimento / Justificativa de Ausência	TCLE_Mielo.docx	02/09/2020 16:17:56	TAMIRIS DA SILVA	Aceito
TCLE / Termos de Assentimento / Justificativa de Ausência	Termo_Ass.docx	18/08/2020 09:46:03	TAMIRIS DA SILVA	Aceito
Folha de Rosto	Folha_de_rosto_Mielo.pdf	11/06/2020 17:28:16	TAMIRIS DA SILVA	Aceito
Projeto Detalhado / Brochura Investigador	Projeto_cep_Tamiris_Mielo.docx	11/06/2020 14:23:10	TAMIRIS DA SILVA	Aceito

Situação do Parecer:
Aprovado

Necessita Apreciação da CONEP:
Não

SAO PAULO, 29 de Setembro de 2020

Assinado por:
Maria Aparecida Dalboni
(Coordenador(a))

Endereço: VERGUEIRO nº 235/249
Bairro: LIBERDADE **CEP:** 01.504-001
UF: SP **Município:** SAO PAULO
Telefone: (11)3385-9010 **E-mail:** comitedeetica@uninove.br
