# Supplementary material for: Effect of photobiomodulation combined with physical therapy on functional performance in children with myelomeningocele: A protocol randomized clinical blind study
Source: PLoS One. 2021 Oct 6;16(10):e0253963. doi: 10.1371/journal.pone.0253963 (PMC8494316; doi:10.1371/journal.pone.0253963)
Supplement: S9 File — (DOCX) [file pone.0253963.s009.docx]

**OPINION SUBSTANTIATED CEP**

# RESEARCH PROJECT DATA

**Research Title:** Effects of physiotherapy associated with photobiomodulation on functional performance in children with myelomeningocele- Clinical, randomized and blind study

**Researcher:** TAMIRIS DA SILVA

# Thematic Area:

**Version:** 3

**CAAE:** 33626720.0.0000.5511

**Institution Proponent:** EDUCATIONAL ASSOCIATION NOVE DE JULHO

**Main Sponsor:** Own Financing

# OPINION DATA

**Opinion Number:** 4,308,134

# Project Presentation:

Information extracted from the project:

Research Title: Effects of physiotherapy associated with photobiomodulation on functional performance in children with myelomeningocele- Clinical, randomized and blind study

Version:3 CAAE:33626720.0.0000.5511

Project Presentation: Summary

Myelomeningocele is a severe type of spina bifida, resulting from inadequate neural tube closure. This condition drastically affects the structures of the spinal cord as a result, the spinal cord, nerve roots and meninges are exposed during pregnancy, resulting in deficiencies. The combination of these deficiencies results in a general decrease in mobility and functional participation. There is little evidence on physical therapy interventions in this population. However, it is known that the International Classification of Functionality, Disability and Health for Children and Young People is a useful tool to assist therapists in problem analysis and reflection on the focus of the intervention. Current literature shows that resources such as low-intensity light, also known as photobiomodulation as a means of

Continuation of the Opinion: 4,308,134

therapeutic, may be auxiliary means in the rehabilitation of neurological conditions, because studies show that photobiomoduction promotes sensory and motor recovery in the animal model of spinal cord injury. And a clinical trial showed that after photobiomodulation treatment combined with photobiomodulation, individuals with spinal cord injury improved motor and sensory function. Therefore, the aim of the study is to evaluate the effects of physiotherapy associated with photobiomodulation on the functional performance of children with low and sacral lumbar myelomeningocele.

Introduction:

Myelomeningocele (MMC) is a severe type of spina bifida, resulting from inadequate neural tube closure.( 1) The multifactorial etiology of CMM is related to environmental and maternal factors. The overall incidence of the disease is one in every 1000 live births. (2) This condition drastically affects the structures of the spinal cord, since the vertebrae at the level of the lesion do not have a spinous process and are therefore incomplete dorsally, this factor causes the spinal cord, nerve roots and meninges to be exposed during pregnancy resulting in deficiencies including paraplegia, skeletal deformities, muscle weakness, loss of sensation, poor coordination, decreased balance, hydrocephalus, Arnold Chiari malformation, and fecal, urinary and sexual dysfunctions. The combination of these deficiencies results in a general decrease in mobility and functional participation. (3) The pathogenesis of CMM is not fully elucidated, but increasing evidence indicates that spinal cord damage associated with CMM occurs due to the primary cause which is related to abnormal spinal cord development and the secondary cause that occurs due to traumatic and chemical injuries subsequent to spinal cord exposure and are associated with loss of neurological function in fetuses with CMM. (4,5) After an injury to the central nervous system (CNS), there is no recovery of most axons due to regenerative failures that occur after CNS damage, which usually induces permanent disabilities. These permanent sequelae occur because astrocytes form scars in order to restrict inflammation and preserve neural tissue, which is an essential process for healing, on the other hand, these chronic scars are harmful because they continuously prevent the regeneration of the axon.( 6) Therefore, the treatment strategies for this condition have the objective of minimizing the extent of the lesion reducing the possible sequelae. The classic treatment for CMM consists of surgical closure of the MMC defect still in the intrauterine phase or shortly after birth. Comparative studies between prenatal and postnatal repair have shown that in both treatments the

Continuation of the Opinion: 4,308,134

functional impairment below the level of the lesion remains incomplete.( 4) Functional impairment was classified by Hoffer et al.( 7) at functional levels according to neurological impairment: thoracic, lumbar high, lumbar low and sacral. The prognosis of ambulation and the objectives to be achieved in rehabilitation depend not only on the neurological level, but also on the presence or not of orthopedic deformities, obesity, cognitive demotion and socio-economic conditions of the family. (8) In relation to physical therapy interventions, there is knowledge that they focus on objectives of optimizing mobility and maximizing independence and participation, which can be facilitated by muscle strengthening, adaptive positioning and / or better postural control. However, evidence on the efficacy of physiotherapy exercises in children with CMM is limited.( 9) It is known that the International Classification of Functionality, Disability and Health for Children and Young People (CIF-CY) is a useful tool to assist therapists regarding problem analysis and thinking about the focus of the intervention. For a long time, the interventions focused on functional deficiencies and the level of body structure, that is, on the child's abilities. More recently, a change has been observed in the literature towards an approach in which interventions focus on environmental factors, that is, the change of restrictions in a task or in the child's environment, thus emphasizing a more efficient way of completing an activity. (10,11) The evidence in physical therapy practice for individuals of all ages with neurological disorders, focuses on 5 Ps: prevention, prediction, participation, personalized and plasticity. Therefore, neurological physiotherapists should perform care with a focus on preventing the patient's disabilities, predicting the ideal response of an intervention, through measures of results of the movement system. In addition, it is of paramount importance that the goal of rehabilitation is that people with neurological disabilities are fully included and participate in life activities that are important to them and that their care is personalized. All these measures facilitate the process of

positive plasticity. (12,13) Neuroplasticity refers to the tendency of neural circuits to suffer

physiological and/or structural changes in response to changes in patterns caused by environmental injuries and/or influences, i.e., there is an increase in angiogenesis and synapses Although neuroplasticity is more commonly associated with the cerebral cortex, all parts of the nervous system, including the spinal cord, demonstrate plasticity, such as synaptic mechanisms of learning and memory, dendritic pruning, proserotation and xonal regeneration. (14) Another factor that influences and regulates neuroplasticity is the neural growth neutron factor (BDNF), the most important functions of BDNF include, regulation

Continuation of the Opinion: 4,308,134

of the syndrome, neuroprotection and increased dendritic afforestation, in addition BDNF influences the functional and structural aspects of the sinaptic transmission. Rehabilitation induces neuroplasticity that can be evidenced by improving the

performance and increased BDNF. (15) The current literature demonstrates that resources that are used as a therapeutic means can be used as auxiliaries in the rehabilitation of neurological conditions, such as stroke, neurodegenerative diseases and spinal cord injuries. Photobiomodulation (PBM) occurs from the application of low intensity light (red and infrared light), such as low-intensity laser and LED in biological tissues. The therapeutic efficacy of Phototherapy is based on the absorption of

photoreceptors or chromophores. (16,17) Studies in the experimental model of spinal cord injury, the authors demonstrated that both red and infrared wavelength have the potential to be effective and noninvasive means of therapy, promoting axonal budding, increased concentration of glial cells and nerve connections, in addition to functional and sensory improvement. (18,19) The findings of a

clinical trial involving individuals with a diagnosis of spinal cord injury demonstrates that PBM exerted positive effects on motor function, especially during isotonic contraction of stimulated muscles evaluated by electromyography (EMG).( 20) In addition, Silva et al. (21) demonstrated that after 12 photobiomodulation sessions associated with physiotherapy in patients with spinal cord injury, there was recovery in

sensory perception and muscle strength. Therefore, FBM may be a promising treatment associated with physical therapy exercises with CMM. Thus, the aim of the study is to evaluate the effects of physiotherapy associated with photobiomodulation on the sensory and motor response of children with low and sacral lumbar myelomeningocele.

Materials and methods:

will be recruited at the Integrated Health Clinic of The Nove de Julho University. Those who meet the inclusion criteria will be randomized to two groups using a randomization site (randomization.com). Group 1 will be submitted to active PBM and physical therapy exercises. Group 2 will be submitted to PBM simulations and physical therapy exercises. Irradiation will be performed with an LED with wavelength of 850 nm, energy per point of 25 J, 50 seconds per point and power of 200

Continuation of the Opinion: 4,308,134

Mw. in the placebo group, the device will not emit light. The objectives of the physiotherapy exercises will be carried out according to the goals and objectives of the child / guardians. the treatment protocol will be twice a week for 24 sessions. assessments will be carried out before treatment at the last treatment session and 30

days after treatment. Muscle activity will be evaluated using a portable electromyography (BTS Engineering) and, as a measure of functionality, the task of sitting and lifting will be performed. The electrodes will be positioned on the muscles: lateral gastrocnemius, anterior tibial and femoral reto. To assess the functional independence of children, the Pediatric Disability Assessment Inventory will be used. Quality of life will be assessed by the Parents' Health Questionnaire - Form 50. The sensory evaluation will be performed with the Kit Semmes- Weinstein (Smiles®). The protein expression of BDNF will be quantified from saliva samples using the ELISA technique. The data will be analyzed with the help of GraphPad PRISM version 7.0.

Chance:

H0: There are no differences in functional performance after treatment with physiotherapy associated with photobiomodulationH1: There are differences in functional performance after treatment with physiotherapy associated with photobiomodulation.

Sample size: 30

# Research Objective:

Primary Objective:

Evaluate the functional performance of children with myelomeningocele after physiotherapy exercises associated with photobiomodulation.

Secondary Objective:

-Evaluate surface sensitivities with Semmes-Weinstein monofilaments;

- Assess functional independence through the PEDI scale;
- Assess quality of life through children's health questionnaire;

– Parents Report 50- Evaluate the protein expression of BDNF levels in saliva samples by ELISA.

Continuation of the Opinion: 4,308,134

Inclusion Criteria:

-age from 5 to 14 years;;

- diagnosis of myelomeningocele at the lower lumbar and sacral level;
- Get Director or Movement to Sit and Stand With Support.

Exclusion Criterion:

- cognitive impairment that compromises the ability to communicate and answer the questions that will be asked;
- latex allergy;
- manifestations secondary to CmM, such as congenital left foot; neuromuscular scoliosis; subluxation or dislocation of the hip and knee;
- another central nervous system disease.

# Risk and Benefit Assessment:

Discomfort or Expected Risks: It may be uncomfortable for the child to lie down for low-intensity light for 5 minutes. Remember that even if minimal, there are always risks. The days of electromyography evaluation may be uncomfortable for the child, because the evaluation time is 30 minutes, and the child may feel impatient. It can also be uncomfortable to collect saliva, because the child needs to spit in the tube.

Research Benefits: The child will perform physiotherapy, the exercises will be according to the functional objective and q main complaint of the child/guardian.

Risk protective measures: The child will be comfortably positioned for low-intensity light. In the collection of saliva the child will spit in a new and sterile tube, in addition professionals will make use of gloves and aprons. All toys used for the development of physiotherapy exercises will be properly cleaned. The light appliance will also be covered with plastic to avoid direct contact with the skin.

Continuation of the Opinion: 4,308,134

# Comments and Considerations about Research:

This is a clinical trial in which 30 children (5 to 14 years of age) with myelomeningocele will be randomized into two groups: (1) photobiomodulation therapy associated with physical therapy exercises and (2) placebo of photobiomodulation associated with physical therapy exercises. The exercises Will be held two sessions per week, totaling 24 sessions. The evaluations will be: muscle activity through da surface electromyography of the lateral gastrocnemius, anterior tibial and reto femoral muscles; functionality through the task of sitting and raising; functional independence through the Pediatric Disability Assessment Inventory; quality of life through the Parents' Health Questionnaire - Form 50; pais; the sensory part through the Semmes-Weinstein kit (Smiles®) and the protein expression of BDNF quantified from saliva samples using the ELISA technique.

The evaluations will be carried out before, after the 24 sessions and one month after the end of the interventions.

# Considerations on the Mandatory Submission Terms:

This project does not present ethical impediments.

The requests for adjustments were met, i.e.:

- The cover page and the basic project information were adequate in previous versions;
- Schedule: Does not specify the year of realization, but the project indicates that the search will start only after approval of the ZIP Code.

The following pending issues were also raised in the first evaluation, with request for:

1- Replace the scientific terms for a language that the layperson can understand: Partially attended - some terms of difficult understanding for the layperson remain, such as "sensory and motor response". Met.

2 - In item 4 (Experimental Phase Procedures), add that if the child misses for 2 consecutive times or has 3 non-consecutive absences should discontinue the intervention - It was not added. Met.

Continuation of the Opinion: 4,308,134

# Recommendations:

It is recommended to correct in the TCLE the phrase:

"The participation of the child may contribute to the expansion of knowledge about myelomeningocele, and the effect of low intensity light associated with physical therapy on muscle strength and sensitivity.", by "The participation of the child may contribute to the expansion of knowledge about myelomeningocele, and the effect of low-intensity light associated with physiotherapy on muscle strength and sensitivity."

# Conclusions or Pending and List of Inadequacies:

All requests were adequately met.

# Final Considerations at cep's discretion:

The researcher must present himself at the research institution (which authorized the study) to begin data collection.

The research participant (or his/her representative) and the responsible researcher must initial all the sheets of the Free and Informed Consent Form - TCLE by placing their signature on the last page of said Term, according to Circular Letter no. 003/2011 of CONEP/CNS.

We emphasize that the researcher should develop the research as outlined in the approved protocol.

Any modifications or amendments to the protocol must be presented to the CEP clearly and succinctly, identifying the part of the protocol to be modified and its justifications. We remind you that this modification will require ethical approval from the ZIP Code before it can be implemented. Objectively with justification for further assessment, the amended documents should be evidenced to facilitate the new analysis.

The researcher is responsible for keeping on file, under his custody, for 5 years, the research data, containing individual forms and all other documents recommended by the CEP (Res. CNS 466/12 item X1. 2. F).

According to Res. CNS 466/12, X.3.b), the researcher must submit to this ZIP Code/SMS the semiannual reports.. The final report must be sent through the Brazil Platform, notification icon. A digital copy of the finalised project should be sent to the body that authorised the

Continuation of the Opinion: 4,308,134

study, via mail, e-mail or delivered in person, as soon as it is completed.

# This opinion was prepared based on the documents listed below:

| Document Type | File | Posting | Author | Situation |
| --- | --- | --- | --- | --- |
| Basic Information  of the Project | PB_INFORMAÇÕES_BÁSICAS_DO_P  ROJETO_1573599.pdf | 02/09/2020  16:18:19 |  | Accepted |
| TCLE / Terms of Nod /  Absence Justification | TCLE_Mielo.docx | 02/09/2020  16:17:56 | TAMIRIS DA SILVA | Accepted |
| TCLE / Terms of  Nod / Absence Justification | Termo_Ass.docx | 18/08/2020  09:46:03 | TAMIRIS DA SILVA | Accepted |
| Cover Sheet | Folha_de_rosto_Mielo.pdf | 11/06/2020  17:28:16 | TAMIRIS DA SILVA | Accepted |
| Detailed Project /  Research Brochure | Projeto_cep_Tamiris_Mielo.docx | 11/06/2020  14:23:10 | TAMIRIS DA SILVA | Accepted |

**Status of the**  **Opinion::**

Approved

# ConeP Needs Appreciation:

No

SAO PAULO, 29 September 2020

# Signed by:

**Maria Aparecida Dalboni (Coordinator)**
